# Long term effects of manual lymphatic drainage and active exercises on physical morbidities, lymphoscintigraphy parameters and lymphedema formation in patients operated due to breast cancer: A clinical trial

**DOI:** 10.1371/journal.pone.0189176

**Published:** 2018-01-05

**Authors:** Mariana Maia Freire de Oliveira, Maria Salete Costa Gurgel, Bárbara Juarez Amorim, Celso Dario Ramos, Sophie Derchain, Natachie Furlan-Santos, César Cabello dos Santos, Luís Otávio Sarian

**Affiliations:** 1 Department of Obstetrics and Gynecology- University of Campinas, School of Medicine, Campinas, São Paulo, Brazil; 2 Department of Nuclear Medicine and Radiology, University of Campinas, School of Medicine, Campinas, São Paulo, Brazil; Academy School of Physical Education, POLAND

## Abstract

**Purpose:**

evaluate whether manual lymphatic drainage (MLD) or active exercise (AE) is associated with shoulder range of motion (ROM), wound complication and changes in the lymphatic parameters after breast cancer (BC) surgery and whether these parameters have an association with lymphedema formation in the long run.

**Methods:**

Clinical trial with 106 women undergoing radical BC surgery, in the Women’s Integrated Healthcare Center—University of Campinas. Women were matched for staging, age and body mass index and were allocated to performed AE or MLD, 2 weekly sessions during one month after surgery. The wound was evaluated 2 months after surgery. ROM, upper limb circumference measurement and upper limb lymphoscintigraphy were performed before surgery, and 2 and 30 months after surgery.

**Results:**

The incidence of seroma, dehiscence and infection did not differ between groups. Both groups showed ROM deficit of flexion and abduction in the second month postoperative and partial recovery after 30 months. Cumulative incidence of lymphedema was 23.8% and did not differ between groups (p = 0.29). Concerning the lymphoscintigraphy parameters, there was a significant convergent trend between baseline degree uptake (p = 0.003) and velocity visualization of axillary lymph nodes (p = 0.001) with lymphedema formation. A reduced marker uptake before or after surgery predicted lymphedema formation in the long run (>2 years). None of the lymphoscintigraphy parameters were shown to be associated with the study group. Age ≤39 years was the factor with the greatest association with lymphedema (p = 0.009). In women with age ≤39 years, BMI >24Kg/m^2^ was significantly associated with lymphedema (p = 0.017). In women over 39 years old, women treated with MLD were at a significantly higher risk of developing lymphedema (p = 0.011).

**Conclusion:**

Lymphatic abnormalities precede lymphedema formation in BC patients. In younger women, obesity seems to be the major player in lymphedema development and, in older women, improving muscle strength through AE can prevent lymphedema. In essence, MLD is as safe and effective as AE in rehabilitation after breast cancer surgery.

## Introduction

Early diagnosis and treatment advances in breast cancer (BC) allowed increased survival [1], so that more women are living with possible side effects of the treatment [2]. Morbidities in the upper limb (UL) ipsilateral to surgery are among the main adverse effects of treatment. One year after surgery, approximately 85% [3] of the women report at least one physical morbidity, such as pain, limited range of motion (ROM), and lymphedema [4]. Therefore, there is an effort to implement preventive measures and treatments to minimize these considerable functional and psychological disturbances [5–7].

Breast cancer related lymphedema is one of the most common physical complications causing substantial functional and psychological disturbance [6,7, 8]. It is a chronic condition that may develop any time after surgery [9]. Recently, questions regarding the role of exercise and the risk of lymphedema have emerged [10]. The benefits of active exercise (AE) in shoulder rehabilitation after breast cancer surgery are extensively described in the literature [11–14], and this approach has become standard practice in referral services [9]. Repercussions of early exercise on the formation of collateral lymphatic vessels and lymphatic flow to promote the prevention of lymphedema are not fully known [14]. Studies showed that women who performed exercises of progressive load had a reduced risk of developing lymphedema [10–15]. Manual lymphatic drainage (MLD) is also widely used in women with lymphedema [16]. Lymphoscintigraphy studies showed that this technique promotes greater absorption of radiopharmaceuticals, possibly indicating a reduced risk for lymphedema [17,18]. However, MLD and AE have not yet been compared long term clinical trials [19,20].

On the other hand, there is no straightforward methodology to predict lymphedema formation, precluding the prescription of preventive measures, such as AE or MLD. Lymphoscintigraphy abnormalities have been found in patients with lymphedema, and the technique has been proposed as an early indicator of lymphedema [18, 21]. In clinical research, lymphoscintigraphy of the upper limb has been used to investigate the association between the anatomy and function of the lymphatic system and the risk of developing lymphedema [22–25], to examine the lymphatic functioning of patients with breast cancer-related lymphedema [26–28], and to evaluate the effect of physical therapy [29,30] or surgical treatment [31] on the lymphatic system.

The aim of this study was thus to compare the long term (up to 30 months after surgery) effects of MLD and AE concerning 1) wound complications, 2) ROM recovery, 3) lymphatic parameters as described by lymphoscintigraphy; and 4) lymphedema formation. To our knowledge, this is the most comprehensive clinical trial assessing the rehabilitation of the upper limb in women with BC.

## Methods

### Subjects

In this clinical trial, we evaluated women undergoing unilateral mastectomy with lymph node dissection due to BC. Women were enrolled between November 2009 and August 2013, and followed-up through 2016. Patients were treated and followed in the Prof. Dr. José Aristodemo Pinotti Women’s Hospital–Women’s Integrated Healthcare Center (CAISM/Unicamp), in Brazil. Women undergoing immediate breast reconstruction were excluded. Also were excluded patients who: (a) before surgery had a difference in UL circumference greater than two centimeters; (b) had motor deficit or infection in the UL ipsilateral to surgery; (c) had undergone radiation therapy; and (d) those unable to understand the exercises proposed (see S1 and S2 Text).

Women who met the inclusion criteria were invited to participate in the study. Those who agreed to participate underwent evaluation at the Physical Therapy Outpatient Clinic of CAISM/Unicamp. One week before surgery, personal and clinical data were collected, UL circumference and goniometry were obtained and lymphoscintigraphy was scheduled. Women were matched for clinical staging, age (±10 years) and BMI, according to the following categories: low weight (<18.5 kg/m^2^); normal weight (≥18.5 and ≤24.9 kg/m^2^); overweight (≥25 and ≤29.9 kg/m^2^); and obesity (≥30 kg/m^2^) and divided into two groups: 52 received MLD and 53 performed active exercises.

**Fig 1:**
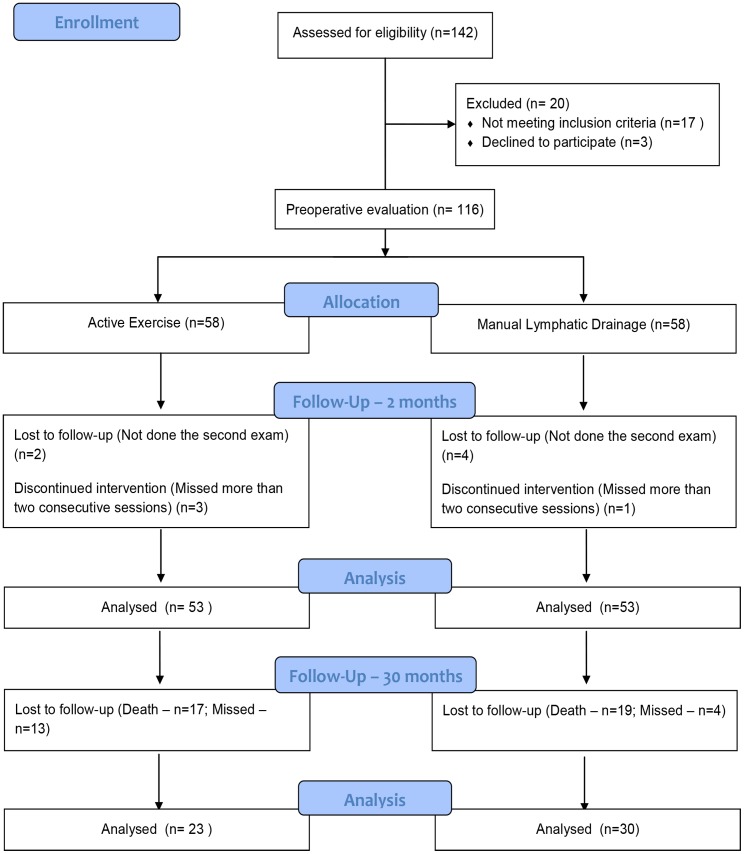
Flowchart of study sample.

Three women from the AE group and one from the MLD group were discontinued because they missed two consecutive treatment sessions (Fig 1).

Sample size was estimated for a controlled non-randomized clinical trial, with a case ratio of 1:1. The prevalence assumed for adequate lymphatic function for the comparison group (exercise) was 52%. Assuming 80% power and 95% confidence intervals (p = 0.05, two-tailed), 48 patients were needed in each group (see S1 and S2 Text).

### Lymphoscintigraphy

Patients underwent lymphoscintigraphy immediately before surgery, 2 and 30 months after surgery at the Nuclear Medicine Service of Unicamp, according to protocol described previously published by a study conducted at our service [23,30].

This analysis includes the velocity visualization of axillary lymph nodes (VVAL) and degree uptake in axillary lymph nodes (DUALN), classified according to Table 1 [23]. The presence or absence of radiopharmaceutical absorption by the liver (RAL), collateral circulation (CC) and dermal backflow (DBFL) were also analyzed. Liver absorption was classified as present when radiopharmaceutical uptake by the liver was visualized in any image; collateral circulation was classified as present when there was drainage of radioactive tracer by a lateral lymphatic vessel or uptake in an epitrochlear lymph node; and dermal backflow was classified as present when there was dermal dispersion of the radiopharmaceutical.

**Table 1:** Visual classification of upper limb lymphoscintigraphy.

Score	Velocity visualization of axillary lymph nodes (VVAL)	Score	Degree uptake in axillary lymph nodes (DUALN)
1	Not visualized	1	Absent
2	After two hours	2	Mild
3	After one hour	3	Moderate
4	At 10 minutes	4	Intense

### Physical evaluation

A physiotherapist evaluation was realized one week before surgery and personal and clinical data were collected. The wound was evaluated 2 months after surgery. ROM, upper limb circumference measurement and upper limb lymphoscintigraphy were performed before surgery, and 2 and 30 months after surgery [11, 30].

### Interventions

#### Educational strategy (both groups)

On the first postoperative day, all participants were provided with information leaflets about proper care for the operated limb to prevent trauma, infection, and to avoid activities that involve too much loading and/or repetition; and daily active exercises to do at home. Furthermore, during the first month after surgery, patients attended lectures delivered by the multidisciplinary team of psychologists, nurses, social service workers and dietitians, composing the Rehabilitation Program for women undergoing breast cancer surgery [11,30] (see S1 and S2 Text).

#### Manual lymphatic drainage (MLD) or active exercise (AE)

Forty-eight hours after surgery, women started the intervention (MLD or AE). Women assigned to MLD group began 40-min individual MLD sessions, twice a week, for 30 days, and those women assigned to the exercise group began 40-min group sessions (5–20 women), twice a week, for 30 days. These techniques were thoroughly described in our previous publications [11,30] (see S1 and S2 Text).

### Statistical analysis

Data were described by calculation of absolute (n) and relative (%) frequencies, mean, median and standard deviation. Group homogeneity was analyzed by Chi-square and Fisher’s exact tests, for nominal and ordinal variables and by student’s t-test and Mann–Whitney tests for quantitative variables. We analyzed whether there was a VVAL and/or DUALN score trend related to the diagnosis of lymphedema and/or the study group using chi-squares for trends. In addition, we analyzed the association of RAL, CC and DBFL with lymphedema and treatment group using chi-squares. Next, we recoded the VVAL and DUALN scores from 1 to 4, and produced an interaction graph showing the mean values of the scores from baseline to 30 months after surgery. Then, we adjusted the analyses for patients’ age and BMI using linear (for VVAL and DUALN) and logistic (for RAL, CC and DBFL) regression models. Finally, we used a recursive partitioning algorithm to confirm the contribution of VVAL DUALN, RDFL, CC and RAL to the prediction of malignant lymphedema formation in up to 30 months postoperatively. Patients’ age, BMI, radio and chemotherapy, smoking habit and treatment (whether AE or MLD) were used as control variables in the recursive partitioning regression model. A conditional inference tree was generated from the model. The recursive partitioning algorithm was based on a linear regression model as described by Hothorn *et al*., to confirm the contribution of the lymphoscintigraphy parameters and control variables to the diagnosis of lymphedema. Conditional inference trees estimate a regression relationship by binary recursive partitioning in a conditional inference framework [32]. Basically, the algorithm works as follows: 1) the global null hypothesis of independence between any of the input variables (lymphoscintigraphy parameters and control variables) and the response (lymphedema) is tested, and the computation stopped if this hypothesis cannot be rejected. Otherwise the input variable with the strongest association to the response is selected. Branches of the generated inference tree bifurcate when a statistically significant association is detected (*P*<0.05). Mann-Whitney and Student’s t-test were used to evaluate the association between shoulder ROM and presence of lymphedema. ANOVA for repeated measures was used to assess the shoulder ROM during follow-up. The software used for analysis was the R Environment for Statistical Computing (R Project). The level of significance adopted was 5%.

### Ethics declaration

The authors confirm that all ongoing and related trials for this intervention are registered. The experiments comply with the current laws of the country in which they were performed. The study was approved by the Research Ethics Committee of Campinas State University (Protocol 1090/2009—from 24^th^ November 2009), according to the Declaration of Helsinki. All patients were informed and signed a free written consent term. Trial registration: Brazilian Registry of Clinical Trials: RBR-3dwp5r (http://www.ensaiosclinicos.gov.br/rg/RBR-3dwp5r/) (see S3 Text).

## Results

Table 2 shows the comparison of the main clinical features of the women in the AE and MLD groups. Of the studied features, only exposure to neoadjuvant chemotherapy was imbalanced between groups: in the AE group, 45.3% of the women received neoadjuvant chemotherapy, contrasted to 67.9% of the women in the MLD group (p = 0.018). The incidence of wound complications did not differ between groups (Table 2).

**Table 2:** Main clinical features of the women in the AE and MLD groups.

	AE		MLD		p-value
	n	%	N	%	
**Age**					0.84
<55 years	22	41.5	24	45.3	
≥55 years	31	58.5	29	54.7	
**Body Mass Index**					0.53
<25 Kg/m^2^	15	28.3	19	35.8	
≥25 Kg/m^2^	38	71.7	34	64.2	
**Type of Surgery**					0.0786*
MRM Patey	29	54.7	19	35.8	
MRM Madden	24	45.3	33	62.3	
RM Halsted	0	0.0	1	1.9	
**Operated breast**					0.8455
Right	29	54.7	28	52.8	
Left	24	45.3	25	47.2	
**Dominant limb**					1.0000*
Right	49	92.2	50	94.3	
Left	4	7.5	3	5.7	
**Clinical Stage**					0.0705*
I	1	2.0	0	0.0	
II	17	34.0	9	18.0	
III/IV	32	64.0	43	82.0	
**Surgical Stage**					0.0915*
I	2	3.8	1	1.9	
II	20	37.7	11	20.8	
III/IV	31	58.5	41	77.4	
**Level of dissected lymph nodes**					0.2703*
1	2	3.8	0	0.0	
2 or 3	51	96.3	53	100.0	
**Neoadjuvant chemotherapy**					0.0187
Yes	24	45.3	36	67.9	
No	29	54.7	17	32.1	
**Adjuvant chemotherapy**					0.0690
Yes	8	36.4	18	62.1	
No	14	63.6	11	37.9	
**Radiotherapy**					0.2901*
Yes	16	72.7	26	86.7	
No	6	27.3	4	13.3	
**Irradiation**					0.1783*
Plastron	1	6.3	6	23.1	
Plastron+SCF	14	87.5	18	69.2	
Plastron+SCF+Axilla	0	0.0	2	7.7	
Plastron+SCF+Cervical region	1	6.3	0	0.0	
**Hormone therapy**					0.790*
Yes	14	63.6	18	60.0	
No	8	36.4	12	40.0	
**Immunotherapy**					1.000*
Yes	3	13.6	5	16.7	
No	19	86.4	25	83.3	
**Shoulderhistory antecedentes**					0.449*
Fracture	1	1.9	5	9.6	
Luxation	1	1.9	0	0.0	
Bursitis	2	3.8	2	3.8	
Tendinitis	5	9.6	5	9.6	
Unknown pain	6	11.5	9	17.3	
Absent	37	71.2	31	59.6	
**Heaviness sensation**					0.3757
Yes	9	36.0	16	64.0	
No	13	48.1	14	51.9	
**Smoking**					1.0000*
Yes	2	40.0	3	60.0	
No	20	42.5	27	57.5	
**Practice of exercises**					0.4575
Yes	8	36.4	14	63.6	
No	14	46.7	16	53.3	
**Care UL**					0.3481*
Follows daily	15	68.2	17	56.7	
Follows partly	5	22.7	12	40.0	
No follows	2	9.1	1	3.3	
**Seroma**					0.2201
Yes	16	40.0	24	60.0	
No	33	52.4	30	47.6	
**Aspiration**					0.1114
Yes	13	37.1	22	62.9	
No	36	53.7	31	46.3	
**Dehiscence**					0.8280
Yes	6	50.0	6	50.0	
No	42	46.7	48	53.3	
**Infection**					0.2723
Yes	15	55.6	12	44.4	
No	32	43.2	42	56.8	

MRM = modified radical mastectomy; RM = radical mastectomy; SCF = supraclavicular fossa; UL = upper limbs/ Chi-Square test;

(*) Fisher’s Exact test

**Fig 2:**
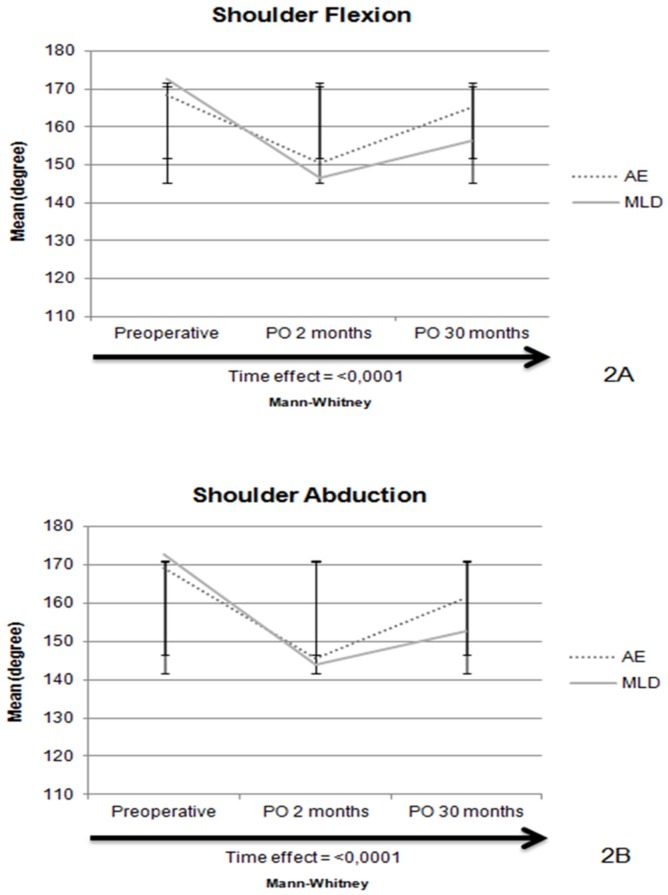
Mean shoulder flexion (2A) and abduction (2B) at the preoperative of surgery, two and thirty months later, according to group.

The mean flexion and abduction ROM in the preoperative evaluation, 2 and 30 months postoperatively were similar in MLD and AE groups. For both groups, UL flexion and abduction were reduced 2 months after surgery compared to baseline, and partial recovery occurred 30 months after surgery. However, there was a significant time effect for shoulder flexion (<0,0001) and abduction (<0,0001) (Fig 2A and 2B).

The cumulative incidence of lymphedema observed was 23.8% and did not differ between groups (p = 0.29) (Data not shown in tables).

In Table 3, we dissect lymphoscintigraphy through its parameters DUALN, VVAL, DBFL, CC and RAL in relation to the cumulative incidence of lymphedema by the end of follow-up (from baseline to up to 30 months postoperatively). All analyses were adjusted for patients’ age and BMI. There was a significant convergent trend between baseline DUALN score (p = 0.003) and VVAL (p = 0.001) with lymphedema formation. The same was true for DUALN at 2 and 30 months (p<0.001 for both analyses), showing that DUALN is a powerful predictor of subsequent lymphedema. In other words, a reduced marker uptake (absent or mild DUALN) before or after surgery predicted lymphedema formation in the long run (>2 years). The DBFL, CC and RAL parameters were not associated with lymphedema formation. In Table 3, we analyse whether the intervention (AE or MLD) was associated with the lymphocintigraphy parameters from baseline to up to 30 months after surgery. None of the studied parameters were shown to be associated with the intervention modality (AE or MLD).

**Table 3:** Lymphocintigraphy parameters DUALN, VVAL, DBFL, CC and RAL as related to cumulative incidence of lymphedema by the end of follow-up.

		Lymphedema			Lymphedema			Lymphedema			Lymphedema			Lymphedema	
		Yes (n = 15)	No (n = 48)		Yes (n = 15)	No (n = 48)		Yes (n = 15)	No (n = 48)		Yes (n = 15)	No (n = 48)		Yes (n = 15)	No (n = 48)
		n(%)	n(%)		n(%)	n(%)		n(%)	n(%)		n(%)	n(%)		n(%)	n(%)
	DUALN			VVAL			DBFL			CC			RAL		
**Baseline**	Intense	4(15)	22(85)	At 10 minute	4 (15)	23 (85)	Present	0	0	Present	3(21)	11(79)	Present	5(21)	19(79)
	Moderate	2(13)	13(87)	After one hours	5(21)	19 (79)	Absent	14(24)	45(76)	Absent	10(23)	34(77)	Absent	10(26)	28(74)
	Mild	4(25)	12(75)	After two hour	1 (17)	5 (83)		P	<0.001		P	1		p	0.842
	Absent	5(100)	0(0)	Not visualized	5 (100)	0(0)									
		p(Trend)	0.003		p(Trend)	0.001									
**2 months**	Intense	2(12)	15(88)	At 10 minute	3(19)	13(81)	Present	1(33)	2(67)	Present	1(8)	12(92)	Present	10(23)	33(77)
	Moderate	1(8)	12(92)	After one hours	3(12.5)	21(87.5)	Absent	12(22)	43(78)	Absent	14(31)	31(69)	Absent	5(26)	14(73)
	Mild	9(39)	14(61)	After two hour	6(46)	7(54)		P	1		P	0.1806		p	1
	Absent	2(33)	4(67)	Not visualized	2(33)	4(67)									
		p(Trend)	<0.001		p(Trend)	0.1115									
**30 months**	Intense	0 (0)	2(100)	At 10 minute	0(0)	2(100)	Present	10(53)	9(47)	Present	6(22)	21(78)	Present	11(24)	35(76)
	Moderate	6(37.5)	15(62.5)	After one hours	6(27)	15(73)	Absent	5(12)	37(88)	Absent	9(26.5)	25(73.5)	Absent	4(27)	11(73)
	Mild	5(33)	10(67)	After two hour	5(38.5)	10(61.5)									
	Absent	4(27)	11(73)	Not visualized	4(27)	11(73)		p(Adjust)	0.975		p(Adjust)	0.105		p(Adjust)	0.998
		p(Trend)	<0.001		p(Trend)	0.1461									

DUALN = degree uptake in axillary lymph nodes; VVAL = velocity visualization of axillary lymph nodes; classified according to Table 1.^11^ The presence or absence of radiopharmaceutical absorption by the liver (RAL), collateral circulation (CC) and dermal backflow (DBFL) were also analyzed

All analyses were adjusted for age and BMI

**Fig 3:**
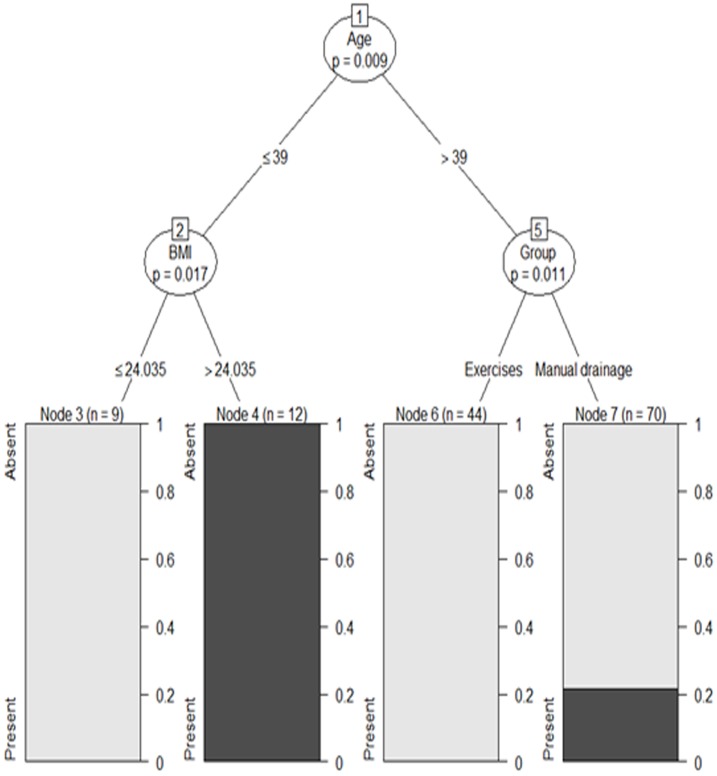
Recursive partitioning showing the factors associated with the presence of lymphedema after 30 months of follow-up.

Table 4 shows the clinical features and postoperative complications as related to lymphedema formation after 30 months of follow-up. None of the studied features or complications was individually associated with lymphedema. Fig 3 shows the result from a partitioning recursive model fit to analyse the clinical factors associated with lymphedema. Clinical factors included in the model were: study group (either AE or MLD), age, BMI, clinical and surgical stage, chemo and radiotherapy and number of dissected lymph nodes. The display shows significant associations at branch bifurcations. Age ≤39 years was the factor with the greatest association with lymphedema (p = 0.009). In women with age ≤39 years, BMI >24Kg/m^2^ was significantly associated with lymphedema (p = 0.017). In women over 39 years old, those who were allotted to MLD group were at a significantly higher risk of developing lymphedema than their counterparts allotted to the AE group (p = 0.011).

**Table 4:** Lymphocintigraphy parameters DUALN, VVAL, DBFL, CC and RAL as related to the intervention group.

		AE	MLD		AE	MLD		AE	MLD		AE	MLD		AE	MLD
		n = 53	n = 53		n = 53	n = 53		n = 53	n = 53		n = 53	n = 53		n = 53	n = 53
	DUALN			VVAL			DBFL			CC			RAL		
**Baseline**	Intense	17(40.5)	25(59.5)	At 10 minute	21(47)	24(53)	Present	0(0)	2(100)	Present	14(58)	10(42)	Present	21(45)	26(55)
	Moderate	16(55)	13(45)	After one hours	10(47.5)	21(52.5)	Absent	47(48.5)	50(51.5)	Absent	31(42.5)	42(57.5)	Absent	32(55)	26(45)
	Mild	12(50)	12(50)	After two hour	5(50)	5(50)		P	0.5202		p	0.2643		P	0.3827
	Absent	8(80)	29(20)	Not visualized	8(80)	2(20)		p(Adjust)	0.224		p(Adjust)	0.148		p(Adjust)	0.360
		p(Adjust)	0.108		p(Adjust)	0.19									
**2 months**	Intense	9(41)	13(59)	At 10 minute	10(42)	14(58)	Present	1(25)	3(75)	Present	9(39)	14(61)	Present	37(51)	35(49)
	Moderate	11(44)	14(56)	After one hours	19(49)	20(51)	Absent	44(47)	49(53)	Absent	40(52)	37(48)	Absent	16(48.5)	17(51.5)
	Mild	16(47)	18(53)	After two hour	7(39)	11(61)		p	0.7157		p	<0.001		P	0.9473
	Absent	8(53)	7(47)	Not visualized	8(53)	7(47)		p(Adjust)	0.491		p(Adjust)	0.374		p(Adjust)	0.412
		p(Adjust)	0.631		p(Adjust)	0.948									
**30 months**	Intense	7(44)	9(56)	At 10 minute	7(64)	4(36)	Present	10(53)	9(47)	Present	17(61)	11(39)	Present	24(51)	23(49)
	Moderate	10(62.5)	6(37.5)	After one hours	9(39)	14(61)	Absent	23(53.5)	20(46.5)	Absent	16(47)	18(53)	Absent	9(60)	6(40)
	Mild	7(47)	8(53)	After two hour	8(61.5)	5(38.5)		p	1		p	0.4141		p	0.759
	Absent	9(60)	6(40)	Not visualized	9(60)	6(40)		p(Adjust)	0.975		p(Adjust)	0.105		p(Adjust)	0.998
		p(Adjust)	0.85		p(Adjust)	0.81									

DUALN = degree uptake in axillary lymph nodes; VVAL = velocity visualization of axillary lymph nodes; classified according to Table 1.^11^ The presence or absence of radiopharmaceutical absorption by the liver (RAL), collateral circulation (CC) and dermal backflow (DBFL) were also analyzed

All analyses were adjusted for age and BMI

On multivariate analysis, no relationship was found between development of lymphedema and flexion and abduction ROM of the UL ipsilateral to surgery in the postoperative evaluations (Table 5).

**Table 5:** Clinical features, postoperative complications and adjuvant treatment as related to the development of lymphedema after 30 months of follow-up.

	Lymphedema				p-value
	Yes		No		
	N	%	n	%	
**Groups**					0.3980
AE	11	30.6	25	69.4	
MLD	9	28.1	23	71.9	
**Age**	20	55.1±17.5	48	59.2±13.0	0.4001
**BMI (kg/m**^**2**^**)**	20	28.07±5.96	48	28.37±5.96	0.7066
**Seroma**					0.4827
Yes	6	33.3	19	40.4	
No	12	66.7	28	59.6	
**Dehiscence**					0.9126*
Yes	3	16.7	6	12.8	
No	15	83.3	41	87.2	
**Infection**					0.2047
Yes	6	33.3	13	27.7	
No	12	66.7	34	72.3	
**Neoadjuvant chemotherapy**					0.5944
Yes	15	75.0	25	52.1	
No	5	25.0	23	47.9	
**Adjuvant chemotherapy**					0.9419
Yes	16	80.0	25	54.3	
No	4	20.0	21	45.7	
**Radiotherapy**					0.9440
Yes	14	82.4	35	76.1	
No	3	17.6	11	23.9	

Chi-Square test;

(*) Fisher’s Exact test;

SCF = supraclavicular fossa; SD = standard deviation

## Discussion

Our study clearly shows that MLD and AE for UL rehabilitation after breast cancer surgery produced similar effects on wound complications, ROM, and lymphoscintigraphy parameters in the short and long run. In addition, our study suggests that AE may be more effective than MLD for the prevention of lymphedema in women older than 39 years. It is also important to mention that the DUALN and VVALN lymphoscintigraphy parameters, when measured before surgery, can help discriminate women at higher risk of developing lymphedema after surgery, regardless of age, body mass index or whether the patient underwent AE or MLD. This prediction power is valid even for the presence of lymphedema in the long term (up to 30 months after surgery).

Studies found that performing exercises in the early postoperative period does not increase the incidence of seroma [33–35] or infection [36,37]. In addition, although MLD improves lymphatic circulation, especially subcutaneous circulation and promotes the removal of interstitial fluid [29], a systematic review found no association between MLD and seroma formation [36].

Our findings show that shoulder flexion and abduction ROM decreased significantly in both groups during the first 60 days after surgery and partial recovery was obtained 30 months after surgery. Previous studies suggest that ROM restriction in the first months may be associated with lymph node dissection, number of dissected lymph nodes and compromised lymph nodes, mastectomy, advanced age, chemotherapy, radiotherapy, hormonal therapy and seroma [38,39].

MLD stimulate alternative drainage pathways, therefore its use in the early postoperative period can reduce the stagnation of proteins in the interstitial space and the risk of increased limb volume resulting from inflammation secondary to surgical damage, which interferes with the lymphatic drainage of the upper limb [17]. In a revision about conservative interventions for preventing lymphedema, Stuiver et al. (2015) [36] demonstrated that the effectiveness of MLD on lymphedema risk is still uncertain. Differences in effect observed in the studies that evaluated the effectiveness of MLD for preventing lymphedema [19,20,40] may in part be ascribed to the methodological differences between studies, e.g. dosage and forms of administration of the MLD [34]. Only three controlled clinical trials studied the MLD effects on the prevention of lymphedema related to breast cancer. Two demonstrated that MLD administered early in the postoperative period can effectively prevent lymphedema [20, 40], whereas the other considered that the addition of MLD did not reduce the incidence of lymphedema [19].

The impact of early AE administration in the formation of new vessels is also discussed [14]. It is believed that regular exercise stimulates lymphangiogenesis, which can reduce the damage caused by dissection and radiotherapy [25]. Lymphoscintigraphy results of women treated for breast cancer may be abnormal even in the absence of signs or symptoms [25]. However, two minutes of exercise normalized lymphatic pressure, accelerating lymph drainage [41]. In sheep with intact lymphatic system, undergoing short duration exercises, there was an increase in the frequency of lymphatic vessels contractions and lymph flow [42]. The exercise causes an increase in blood pressure and cardiac output, resulting in increased capillary filtration, which leads to an increase in interstitial pressure, facilitating entry of fluid and proteins into the lymph capillaries. Other authors observed that, during exercise, both mechanisms, intrinsic and extrinsic, are reinforced, increasing the propulsion of the lymph through the lymph vessels [26,27]. Regardless of what happens to the lymphatic vessels, the positive impact of exercise on muscle and cardiovascular systems seems to favor the removal and circulation of lymph [43].

As mentioned above, the mechanisms through which AE and MLD exert their effects on the lymphatic system are distinct. However, our study shows that their end results are similar, including lymphoscintigraphy parameters and prevention of clinically detectable lymphedema. Recently, a study with breast cancer patients scheduled for modified radical mastectomy randomly apportioned to undergo physical exercise only (n = 500) or self-MLD as well as exercise (n = 500) after surgery and followed for 12 months showed that self-MLD, in combination with physical exercise, is beneficial for breast cancer patients in preventing postmastectomy scar formation, upper limb lymphedema, and shoulder joint dysfunction [44]. It seems that in older people, lower physical activity, weakness of muscle pump, and higher BMI may increase edema [5]. This in turn may help explain our finding that older women benefited more from AE than MLD, since AE may have helped improve muscle strength.

Swelling may occur at any point following axillary node dissection or radiation therapy, beginning immediately after or even delayed by several years [5] and the incidence seems to increase up to 2 years after diagnosis or surgery of breast cancer [2]. Our findings revealed that lymphatic drainage pattern alterations were not related to the presence of signs during the first 2 months after surgery, while after 30 months, 23.9% of the women evaluated showed lymphedema. Meta-analysis of nine prospective cohort studies gives an incidence of lymphedema diagnosed by more than one method of 28.2% (mean; range 11.8%–53.5%) [2].

The existing data on whether lymphedema incidence varies with age is inconsistent [45]. Hayes showed that age more than 50 may increase the risk of lymphedema incidence 3.3 times [46]. In addition, object arm circumferential measures are significantly higher in women >55years old compared to those made in younger patients [47]. However, our findings showed that lymphedema was associated with younger age at diagnosis and the same was observed in other studies [5,8,21]. The reason why older women are less likely to develop lymphedema later in the survival trajectory is unclear [45]. Reports show that older women who survived breast cancer are more likely to attribute their symptoms to aging or other comorbidities, whereas younger women, especially those who are active in work or engaged in recreational/parenthood activities, may be more aware of the limitations imposed by lymphedema [21].

We also detected an association of lymphedema risk and higher BMI. Although obesity is a major clinical risk factor for lymphedema, the mechanisms that regulate this effect remain unknown [48,49]. Recent reports have demonstrated that obesity is associated with acquired lymphatic dysfunction [48,50]. The hypothesis to explain obesity-induced lymphedema are that the lymphatics in the extremity are normal but the elevated pressure from the weight of the tissue or progressive skin folds also might collapse lymphatic vessels [49]; or yet to impaired baseline lymphatic clearance and an increased propensity for inflammation [48], fibrosis and adipose deposition may destroy lymph vessels and/or nodes [5,48–50].

In the current study, a considerable number of women from both groups showed radiopharmaceutical VVALN and DUALN decrease in postoperative lymphoscintigraphy. This worsening occurred regardless of the rehabilitation technique employed and may be related to lymphatic system impairment caused by axillary dissection, surgical aggressiveness [6], altered scar formation and presence of seroma [51], as well as the preoperative constitution of lymphatic system [28,52]. This later effect may help explain why VVALN and DUALN parameters before surgery were associated with lymphedema formation after surgery. In this same line of thought, Cintolesi et al. observed that in women destined to develop lymphedema the lymphatic contractile work rate is already high and if this is raised further by increased resistance to axillaries drainage following cancer treatment and/or further increase in lymph load, the lymphatics are gradually tipped into chronic failure. The authors suggest an analogy to high preload and high after load cardiac failure in systemic hypertension, where the reduced lymphatic pump activity then leads to overt clinical edema [52].

In essence, our study shows that manual lymphatic drainage is as safe and effective as exercise in rehabilitation after breast cancer surgery. Also of note, our data clearly shows that lymphatic abnormalities precede lymphedema formation in BC patients. In younger women, obesity seems to be the major player in lymphedema development, and actions devoted to reduce body weight may be of great benefit to women undergoing surgery for BC. On the other hand, our study shows that in older women, interventions focused on improving muscle strength are pivotal in preventing lymphedema formation.

Studies have suggested the use of Kinesiotaping, extracorporeal shock wave therapy and low-level laser therapy in the treatment of lymphedema. Among the physiological effects of these resources are the improvement of microcirculation, activation of lymph drainage [53,54,55,56], increased lymphangiogenesis [54,55,56], and decreased neutrophils, inflammation [54,55] and adipocyte count [54]. In this way, further research can be carried out to provide evidence of preventive effects of these resources in lymphedema formation.

### Study limitation

We believe that our study shows evidence that the MLD and AE effects are equivalent regarding postoperative morbidities in patients treated for BC. Our study, however, has some limitations. First, the study design is suboptimal, since this is not a randomized controlled trial. On the other hand, careful balancing of baseline characteristics and multivariate analyses were used to compensate for possible selection biases. Second, we have not included women who underwent sentinel lymph node biopsy, which is the current standard of care for many clinical situations. We acknowledge that this is a limiting analytical factor. Other exclusion criteria, such as previous radiotherapy and breast reconstruction, in our view, bring more benefits than detrimental effects to the analysis.

## Conclusion

MLD is as safe and effective as exercise in rehabilitation after breast cancer surgery. Both techniques have the same effect on ROM, wound, lymphatic parameters as described by lymphoscintigraphy and lymphedema incidence up to 30 months after surgery. Impaired absorption and lymph flow evaluated preoperatively by lymphoscintigraphy are predictors for the development of lymphedema in the long term.

## Supporting information

S1 Text Final project. (DOC)

S2 Text Original study protocol. (DOC)

S3 Text CONSORT 2010 checklist. (DOC)
