# Supplementary material for: Long term effects of manual lymphatic drainage and active exercises on physical morbidities, lymphoscintigraphy parameters and lymphedema formation in patients operated due to breast cancer: A clinical trial
Source: PLoS One. 2018 Jan 5;13(1):e0189176. doi: 10.1371/journal.pone.0189176 (PMC5755747; doi:10.1371/journal.pone.0189176)
Supplement: S1 Text — (DOC) [file pone.0189176.s001.doc]

**COMPARAÇÃO DA CINESIOTERAPIA E DA DRENAGEM LINFÁTICA MANUAL NAS COMPLICAÇÕES, COMPENSAÇÕES LINFÁTICAS E REABILITAÇÃO DO OMBRO NO PÓS-OPERATÓRIO DE CÂNCER DE MAMA**

**Projeto de Doutorado - Junho de 2009**

**Pesquisador responsável:** Mariana Maia Freire de Oliveira

**Função:** Fisioterapeuta (Possui currículo Lattes)

**Orientadora:** Profª. Drª. Maria Salete Costa Gurgel

**Função:** Professor Associado (Possui currículo Lattes)

**Pesquisadores colaboradores:**

**Dra. Bárbara Juarez**

**Função:**

**Dr.**

**Função: Residente da Medicina Nuclear**

Autorizo a realização desta pesquisa na Divisão de Oncologia Ginecológica e Patologia Mamária do CAISM/UNICAMP.

____________________________

Prof. Dr. Gustavo Antônio de Souza

Autorizo a realização desta pesquisa no Serviço de Medicina Nuclear do Departamento de Radiologia – FCM - UNICAMP.

______________________________

Prof. Dr. Nelson Caserta

Autorizo a realização desta pesquisa na Seção de Fisioterapia do CAISM -UNICAMP.

_______________________________

Profa. Ms. Regina Coppo

**Pesquisador responsável:** Mariana Maia Freire de Oliveira

**RG:** M – 8.678.064

**CPF:** 039.024.496-16

**Função:** Fisioterapeuta

**Telefones para Contato:** Residência: 3342-1709 / Celular: 8155-6031

**E-mail:** marimfo@yahoo.com.br

**Orientadora:** Profª. Drª. Maria Salete Costa Gurgel

**RG:** 38.542.836-4

**CPF:** 025.026.918-00

**Função:** Professor Associado

**Local de Trabalho:** DTG – FCM – UNICAMP

**Telefone para Contato:** Residência: (19) 3287-5984

Celular: (19) 9113-9057 - Trabalho: (19) 3521-9305

**E-mail:** salete@caism.unicamp.br

**Pesquisadores colaboradores:**

**Dra. Bárbara**

**RG:**

**CPF:**

**Função:**

**Telefones para Contato:**

**E-mail:**

**Residente da Medicina Nuclear**

**RG:**

**CPF:**

**Função:**

**Telefones para Contato:**

## **E-mail:**

## **RESUMO**

O linfedema de membro superior ipsilateral à mama mastectomizada apresenta incidência entre 24 e 49% e pode estar presente imediatamente após a cirurgia ou anos após o tratamento, promovendo substancial prejuízo funcional e psicológico para a paciente. Vários são os fatores relacionados ao desenvolvimento do linfedema: esvazimento axilar, radioterapia, nível de retirada dos linfonodos, extensão da técnica cirúrgica dentre outros. **Objetivo:** Avaliar o efeito da cinesioterapia e da drenagem linfática manual (DML) nas complicações, compensações linfáticas e na reabilitação do ombro no pós-operatório de câncer de mama. **Sujeitos e métodos:** Será um ensaio clínico randomizado para o qual serão convidadas mulheres que serão submetidas a tratamento cirúrgico radical por câncer de mama no Centro de Atenção Integral à Saúde da Mulher da Universidade Estadual de Campinas. As que aceitarem serão alocadas aleatoriamente em dois grupos: o grupo 1 - cinesioterapia (n=40) e o grupo 2, DLM (n=40). Os dados sócio-demográficos e clínicos e a avaliação física serão coletados por ocasião da avaliação pré-operatória e a reavaliação será feita 30 e 60 dias após a cirurgia no Ambulatório de Fisioterapia. As compensações linfáticas serão avaliadas pelo exame de linfocintilografia que será realizado no Hospital das Clínicas da UNICAMP, sendo um exame pré-operatório e outro 60 dias após a cirurgia. Todos os dados serão obtidos através do laudo de cada exame, que segue os padrões da ficha de coleta de dados. Os dados serão avaliados descritivamente através do cálculo de freqüências absolutas (n) e relativas (%) para variáveis categóricas e através de média, mediana e desvio-padrão para as variáveis contínuas. A homogeneidade dos grupos será analisada através do teste de Qui-quadrado ou Exato de Fisher, para variávies nominais e ordinais e do T-Student ou Mann-Whitney para as variáveis discretas. Para a comparação da evolução das medidas de ângulos e demais variáveis contínuas nos grupos será utilizado a MANOVA para medidas repetidas. O nível de significância assumido no trabalho é de 5%.

- SUMÁRIO
- Introdução .........................................................................................................01
- Justificativa........................................................................................................11
- Objetivos ...........................................................................................................12
- Objetivo geral ...............................................................................................12
- Objetivo específico .......................................................................................12
- Hipóteses ..........................................................................................................13
- Sujeitos e Métodos ...........................................................................................13
- Desenho ......................................................................................................13
- Tamanho amostral ........................................................................................13
- Seleção de sujeitos .......................................................................................14
- 5.3.1. Critérios de inclusão .............................................................................14
- 5.3.2. Critérios de exclusão ............................................................................14

Variáveis ....................................................................................................14

- 5.4.1. Variável independente ..........................................................................14
- 5.4.2. Variável dependente .............................................................................22
- 5.4.3. Variável de controle ..............................................................................16

Técnicas, testes e/ou exames ....................................................................17

- 5.5.1. Técnica ..................................................................................................17

5.5.2. Testes ...................................................................................................19

Instrumento para coleta de dados .............................................................21

Coleta de dados .........................................................................................21

Acompanhamento dos sujeitos ..................................................................21

- Controle de qualidade.......................................................................................22
- Processamento e análise dos dados ................................................................22
- Processamento .............................................................................................22

Análise ........................................................................................................22

Apresentação dos dados...................................................................................23

- Aspectos éticos ................................................................................................25
- 10. Referências bibliográficas ...............................................................................26

11. Cronograma......................................................................................................30

12. Orçamento.. .....................................................................................................31

ANEXO 1 ...............................................................................................................50

ANEXO 2 ...............................................................................................................55

**1. Introdução**

O câncer de mama apresenta grandes e significativos índices de mortalidade entre as mulheres, sendo que, no Brasil, dos 234.870 novos casos de câncer com diagnóstico previsto para 2008, o câncer de mama foi o segundo mais incidente entre a população feminina, sendo responsável por 49.400 novos casos ^1^. Considerando a expectativa de sobrevida de 17,5 anos para essas mulheres, tornou-se fundamental o aprimoramento das técnicas de reabilitação para proporcionar-lhes qualidade de vida física e mental ^2^.

O linfedema de membro superior ipsilateral à mama operada, apresenta incidência entre 24 e 49% ^3^ e pode estar presente imediatamente após a cirurgia ou anos após o tratamento. No entanto, a maioria dos casos ocorre durante os primeiros 18 meses ^4,5,6^ promovendo um substancial prejuízo funcional e psicológico para a paciente.

Os fatores relacionados ao desenvolvimento do linfedema são o esvazimento axilar e a radioterapia ^7, 8^, idade, número de linfonodos dissecados, número de linfonodos positivos, nível de retirada dos linfonodos ^9,10^ obesidade, extensão da técnica cirúrgica e infecção ^3,11^.

O linfedema é um problema quantitativo entre o fluxo linfático produzido e a capacidade de transporte. Se a produção normal de proteínas linfáticas for maior que a capacidade de transporte, o linfedema aparecerá imediatamente ^12, 13, 14^.

A redução do transporte de linfa no braço e no quadrante superior do tronco oferece maior risco de infecção, fibrose, síndrome compartimental e morbidades físicas como alterações da pele, diminuição da sensibilidade e função do membro e dor com variada intensidade e freqüência ^15^.

O sistema linfático possui várias propriedades importantes entre elas o controle da homeostase macromolecular, absorção de lipídeos, metástases, função imunológica e controle dos fluidos teciduais ^16,17,18^. Na tentativa de suprir a ausência dos linfonodos retirados, adequando assim a capacidade de transporte da linfa, após a dissecção axilar o sistema linfático buscará mecanismos de compensação, tais como a ativação de vasos remanescentes, anastomoses axilo-axilares e axilo-inguinais, linfo-linfáticas na região axilar e linfo-venosas periféricas, migração de fluído rico em proteínas em direção aos vasos saudáveis e maior atividade dos macrófagos ^14^.

A linfangiogênese reduz o estresse nos danos nos vasos linfáticos decorrente da dissecção axilar ^19^ e, em muitos casos, a capacidade de transporte dos novos vasos formados parece suficiente para prevenir a manifestação clínica do edema. A regeneração dos vasos linfáticos interrompidos através das anastomoses linfo-linfáticas pode ser prejudicada pela alterada formação cicatricial, seroma pós-operatório, radioterapia e exercícios precoces não adequados para reabilitação do ombro ^14^. Tecidos menos prejudicados em cirurgias menos agressivas permitem novas conexões linfo-linfáticas ^20^.

Em um estudo experimental com animais foi possível observar que após a retirada do linfonodo houve, em um período de quatro semanas, o aparecimento de um plexo de pequenos vasos como uma ponte lacunada de ligação entre o ducto pré-nodal e os vasos pré-nodais, sem evidência clínica de edema no membro do animal, sugerindo que a drenagem linfática superficial tenha sido restaurada. Entretanto, essas pontes apresentaram estruturas muito irregulares e se enredaram no tecido fibroso, distorcendo o caminho do vaso linfático o que gerava um aumento da resistência do fluxo linfático. Dessa forma, da geração de novos vasos linfáticos, houve prejuízo para o fluxo de transporte da linfa ^13^.

A restauração do fluxo linfático é beneficiada pela boa cicatrização tecidual, pela imobilização do ombro ipsilateral à cirurgia, pela drenagem linfática manual (DLM) e pela contração muscular ^14,18,20^. No entanto, a imobilização do ombro favorece o acúmulo de proteínas no interstício, predispondo a um maior risco de fibrose tecidual ^20^.

A resposta fisiológica do exercício envolve aumento na freqüência cardíaca, ativação do sistema linfático, contração de musculatura esquelética e respiração, que constituem mecanismos de auxílio na formação e propulsão linfática ^19^. Além disso, a realização de exercícios regulares (cinesioterapia) resulta na criação de novas vias de drenagem linfática para os linfonodos supraclaviculares do lado afetado^19^, sugerindo as vantagens do início precoce dos exercícios na recuperação física da mulher ^16,21,22^.

Esta abordagem é extensamente descrita na literatura ^23-,25^, sendo adotado como protocolo em serviços de referência ^23-26^. No entanto, estudos não esclarecem a repercussão dos exercícios precoces na formação dos vasos colaterais e sobre o fluxo linfático. Sabe-se apenas que durante o exercício há um aumento da pressão dos vasos linfáticos em humanos, em uma freqüência de 2,4+0,5/min no repouso para 5,4+0,7/min durante o exercício ^18^.

A drenagem linfática manual (DLM) aumenta o fluxo linfático sem aumentar a filtração capilar ^27^. Entre seus efeitos fisiológicos encontram-se: aumento na contração dos linfangions ^28^, aumento da absorção de proteínas pelos capilares linfáticos ^29^, redução da hipertensão microlinfática ^30^ e melhora da drenagem linfática colateral entre as regiões linfáticas da pele ^31,32^. Estudos concordam que a drenagem linfática manual realizada após a linfadenectomia conduz a diminuição da circunferência do membro ^33^ e possibilita maior absorção do radiofármaco (53,2%) nos linfonodos visualizados previamente a DLM, assim como a visualização de outros linfonodos não evidentes ^32^.

A heterogeneidade das técnicas cirúrgicas, dos grupos de pacientes, das variáveis avaliadas e das formas de mensuração resulta em controvérsias quanto aos efeitos benéficos dos exercícios. Embora largamente utilizada, a drenagem linfática manual no pós-operatório de câncer de mama é pouco estudada. Desta forma, o impacto da reabilitação física no pós-operatório de câncer de mama sobre as possíveis complicações na articulação do ombro, do tecido e da função linfática de curto e longo prazo, constitui uma questão importante.

Pesquisas que investigam a eficácia de intervenções terapêuticas para tratar e/ou prevenir o linfedema são limitadas ao uso de medidas antropométricas, como a perimetria e volumetria. Tais exames são simples e não invasivos, mas não informam mudanças na fisiologia do sistema linfático. A linfocintilografia acrescenta outra dimensão na avaliação do sistema linfático ^18^.

A linfocintilografia tem sido usada desde a década de 1950 para estudar patologias do sistema linfático, e atualmente é defendida como principal teste diagnóstico para o sistema linfático periférico ^9^. Apresenta aplicação clínica para indicar e quantificar a drenagem linfática sob os pontos de vista morfológico e funcional, determinar o número de linfonodos sentinelas e identificar pacientes de risco para desenvolvimento de linfedema após esvaziamento da axila ^34,35^. É um método confiável para mensurar o fluxo linfático em condições incertas de aumento ou redução dessa atividade ^9,36,37^.

Considera-se fluxo anormal do sistema linfático a falta da trilha de migração e a lentidão no transporte do radiofármaco, o fluxo contrário do fluido para a derme, a presença de largos vasos linfáticos, a não-visualização dos vasos linfáticos do lado envolvido, a ausência ou pobre visualização dos linfonodos remanescentes e/ou detectáveis ^34,38^. Portanto, a linfocintilografia realizada antes e após a dissecção axilar fornece informações importantes que podem predizer o desenvolvimento de condições patológicas, como o linfedema ^39^.

Além de diagnosticar e categorizar a severidade do linfedema, a linfocintilografia pode ser empregada para o resultado do seu tratamento e identificar o risco para seu desenvolvimento antes que qualquer manifestação clínica esteja presente ^39,40^.

Em recente estudo realizado no CAISM/UNICAMP encontrou-se alterações relevantes nas linfocintilografias pré e pós-operatórias em mulheres mastectomizadas, demonstrando a existência de diferenças funcionais do sistema linfático do membro superior ^41^.

Foram estudadas 37 pacientes que realizaram linfocintilografia de membro superior até 30 dias antes da mastectomia no período de setembro de 2006 a junho de 2007. O exame consistiu na realização de imagens estáticas do membro superior em semi-flexão após 10 minutos, 1 e 2 horas da injeção subcutânea de dextran-^99m^Tc no dorso da mão. Quatro (11%) pacientes apresentaram o padrão de estado da funcionalidade linfática considerado ideal (linfonodos axilares visíveis aos 10 minutos e captação acentuada do contraste) no membro superior ipsilateral enquanto seis (19%) apresentaram o mesmo padrão no membro contralateral; três (8%) pacientes apresentaram a pior classificação (linfonodos axilares não visíveis e ausência de captação do contraste) no membro superior ipsilateral e duas (6%) no contralateral; as demais apresentaram estados intermediários de velocidade e intensidade de captação. Esse estudo encontrou relevantes alterações na linfocintilografia pré-operatória, demonstrando a pré-existência de diferenças funcionais do sistema linfático ^41^.

Vinte e três destas 37 pacientes foram acompanhadas tendo repetido a avaliação linfocintilográfica após 60 dias de pós-operatório. Três (13%) pacientes foram consideradas com o padrão ideal (Ia) e duas (9%) apresentaram total comprometimento (IVd). Quanto à velocidade de captação do contraste, 9 (39%) não apresentaram diferença, 5 (22%) melhoraram e 9 (39%) pioraram, sendo uma com piora acentuada. Quanto à intensidade de captação, 10 (43%) pacientes não apresentaram diferença, 9 (39%) pioraram e 4 (17%) melhoraram. Esse estudo encontrou relevantes alterações nas linfocintilografias pré e pós-operatórias, demonstrando a existência de diferenças funcionais do sistema linfático do membro superior. Alterações no padrão de drenagem linfática já podem ser percebidas com 60 dias de pós-operatório, assim como o indício de presença de anastomose linfo-venosa ^41^.

Conhecer a repercussão da cinesioterapia e da drenagem linfática manual para a reabilitação física sobre os mecanismos de compensação linfáticos, sobre a amplitude de movimento do ombro e sobre as compensações/complicações linfáticas trará benefícios para as mulheres submetidas à cirurgia por câncer de mama com esvaziamento axilar. Sendo possível analisar e comparar os resultados encontrados, essas mulheres poderão participar de programas de fisioterapia que proporcionem melhores resultados na reabilitação do ombro, menores incidências de complicações no pós-operatório imediato e de programas de prevenção de linfedema mais eficazes e objetivos, minimizando a incidência dessa complicação.

**2. Justificativa**

Os exercícios no pós-operatório de câncer de mama estão bem estabelecidos na literatura como forma de recuperação das pacientes para as atividades funcionais diárias, laborais e de lazer. Entretanto, o sistema linfático de membro superior homolateral à cirurgia está sujeito a alterações decorrentes da linfonodectomia axilar aumentando o risco de linfedema. A associação entre a realização da drenagem linfática manual (DLM) à normalização do padrão linfático ainda é pouco explorada.

Esse estudo tem como objetivo observar como os exercícios e a DLM realizados no pós-operatório para câncer de mama influenciam as compensações do sistema linfático e a recuperação funcional do ombro homolateral à cirurgia. Dessa maneira será possível esclarecer se os exercícios e/ou a DLM podem minimizar o aparecimento de complicações pós-operatórias. Além disso, permitirá melhorar o conhecimento dos mecanismos de compensação linfática após a realização dos exercícios e da DLM fornecendo informações fundamentais ao estabelecimento de ações preventivas para o linfedema, contribuindo para o aprimoramento dos programas de reabilitação física nos serviços de fisioterapia.

**3. Objetivos**

**3.1. Objetivo geral**

Avaliar o efeito da cinesioterapia e da drenagem linfática manual nas complicações, compensações linfáticas e na reabilitação do ombro no pós-operatório de câncer de mama.

**3.2 Objetivos específicos**

Comparar, entre o grupo de mulheres que realizou cinesioterapia e o que realizou DLM:

- tempo total de permanência do dreno;
- incidência de seroma após 60 dias de cirurgia;
- tempo de aparecimento do radiofármaco nos vasos linfáticos e nos linfonodos regionais no pré-operatório e após 60 dias da cirurgia;
- presença de circulação colateral e de refluxo dérmico após 60 dias da cirurgia;
- amplitude dos movimentos da articulação do ombro no pré-operatório, 30 dias e 60 dias da após a cirurgia;
- circunferência do membro superior homolateral no pré-operatório, 30 dias e 60 dias da após a cirurgia.

**4. Hipóteses**

**4.1.** O tempo total de permanência do dreno será menor no grupo de mulheres que realizou DLM.

**4.2.** A incidência de seroma será menor no grupo de mulheres que realizou DLM.

**4.3.** O tempo de aparecimento do radiofármaco nos vasos linfáticos e nos linfonodos regionais ao final dos 60 dias será menor no grupo de mulheres que realizou DLM.

**4.4.** A presença de circulação colateral e de refluxo dérmico ao final dos 60 dias será menor no grupo de mulheres que realizou DLM.

**4.5.** A amplitude de movimento (ADM) do ombro aos 30 e 60 dias de pós-operatório será maior no grupo que realizou exercícios.

**4.6.** A circunferência do membro superior ao final dos 30 e 60 dias será menor no grupo de mulheres que realizou DLM.

**5. Sujeitos e métodos**

**5.1. Desenho do estudo**

Será realizado um ensaio clínico controlado randomizado.

**5.2. Tamanho amostral**

Os escassos estudos referentes à análise da influência dos exercícios ou drenagem linfática manual na função linfática, avaliada pela linfocintililografia, de membros superiores de mulheres submetidas à cirurgia por câncer de mama seguem formas de mensuração heterogêneas. Desta forma, foi definido que os dados serão coletados por um período de um ano. Estima-se que serão estudadas 80 mulheres, randomizadas 40 em cada grupo de tratamento.

Segundo dados do Controle de Informações Cirúrgicas do CAISM/UNICAMP no período de 01 de janeiro de 2008 a 31 de dezembro de 2008, estima-se que neste período aproximadamente 120 mulheres obedeçam aos critérios de inclusão, possibilitando alcançar a amostra definida.

**5.3. Seleção dos sujeitos**

Serão selecionadas para participar desse estudo mulheres com câncer de mama que serão submetidas à mastectomia radical, no CAISM-UNICAMP. As mulheres que preencherem os critérios abaixo explicitados serão convidadas, no quando do agendamento da cirurgia, pela pesquisadora responsável a participar do estudo. Neste mesmo momento, serão alocadas nos grupos de cinesioterapia ou DLM, através de tabela de aleatorização gerada por um programa computadorizado.

**5.3.1. Critérios de inclusão**

- Primeira cirurgia por carcinoma invasivo de mama ipsilateral;
- Realização de mastectomia radical.

**5.3.2. Critérios de exclusão**

- Reconstrução mamária imediata;
- Cirurgia bilateral;
- Diferença na circunferência dos membros superiores prévia à cirurgia maior que dois centímetros;
- Deficiência motora prévia em membro superior homolateral à cirurgia;
- Infecção prévia no membro superior homolateral à cirurgia prévia;
- Realização de biópsia de linfonodo sentinela;
- Realização de radioterapia prévia à cirurgia;
- Incapacidade de compreender os exercícios propostos.

**5.4. Variáveis**

A seguir são definidas as variáveis que serão estudadas e apresentadas suas categorias.

**5.4.1. Variável independente**

Técnica fisioterapêutica utilizada para reabilitação pós operatória categorizada em:

- Cinesioterapia - movimento ativo-livre dos ombros, realizado pelo sujeito, segundo protocolo previamente definido e sob orientação de um fisioterapeuta, a ser categorizado em realizado ou não realizado.
- Drenagem linfática manual - Técnica de massagem que visa o aumento da atividade linfocinética nos canais linfáticos normais e a motricidade do linfagion, além do relaxamento e/ou amolecimento do tecido conjuntivo alterado, a ser categorizado em realizado ou não realizado.

**5.4.2. Variáveis dependentes**

- Tempo de permanência do dreno, em dias.
- Seroma – presença de secreção coletada no sítio operatório necessitando aspiração por punção depois de retirada do dreno, categorizada em sim ou não.

## ADM de Flexão de ombro - capacidade total de flexão do ombro feito voluntariamente pelo sujeito no plano sagital em torno do eixo medio-lateral. Será medida com o auxílio do goniômetro em decúbito dorsal, no pré-operatório e 60º dias de pós-operatório: em graus.

- ADM de Abdução de ombro - capacidade total de abduzir o ombro feita voluntariamente pelo sujeito no plano frontal ao redor do eixo ântero-posterior. Será medida com o auxílio do goniômetro em decúbito lateral, no pré-operatório, 30º e 60º dias de pós-operatório: em graus.
- Circunferência do membro superior ipsilateral à cirurgia – mensuração da circunferência no membro superior. Será medida com auxílio de uma fita métrica flexível, medido no pré-operatório, 30º e 60º dias de pós-operatório: em centímetros.
- Tempo de aparecimento do radiofármaco nos vasos linfáticos – tempo de aparecimento do fitato-99mTc após a injeção subcutânea nos vasos linfáticos, avaliado através da linfocintilografia após 60 dias da cirurgia: presente em 10 minutos/ presente em 1 hora/ presente em 2 horas/ ausente.
- Tempo de aparecimento do radiofármaco nos linfonodos regionais – tempo de aparecimento do fitato-99mTc após a injeção subcutânea nos linfonodos axilares, avaliado através da linfocintilografia no pré e após 60 dias da cirurgia: presente em 10 minutos/ presente em 1 hora/ presente em 2 horas/ ausente.
- Presença de circulação colateral – presença de vasos linfáticos neoformados após 60 dias da cirurgia, avaliado através da linfocintilogragia: presente em 10 minutos/ presente em 1 hora/ presente em 2 horas/ ausente.
- Presença de refluxo dérmico – presença de fluxo linfático retrógrado para a pele após 60 dias da cirurgia, avaliado através da linfocintilogragia: presente em 10 minutos/ presente em 1 hora/ presente em 2 horas/ ausente.

**5.4.3. Variáveis de controle**

- Tipo de cirurgia - diferentes técnicas cirúrgicas utilizadas: mastectomia radical tipo Pattey, Madden ou Halsted.
- Idade - número de anos completos na primeira entrevista, segundo relatado pelo sujeito.
- Número de linfonodos comprometidos – número de gânglios com células neoplásicas histologicamente confirmadas, encontrados após a ressecção axilar.
- Número de linfonodos dissecados – número de gânglios retirados na ressecção axilar.
- Estadiamento cirúrgico – avaliação da extensão anatômica da doença e dos órgãos acometidos, realizada depois da cirurgia – EpI, pIIa, pIIb, pIIIa, pIIIb, pIIIc e pIV, segundo os critérios da AJCC (Singletary et al, 2002).
- Índice de massa corpórea – peso dividido pela altura ao quadrado, medida na internação, em Kg/m^2^.
- Infecção da ferida cirúrgica – secreção de aspecto purulento na ferida cirúrgica, segundo conste no prontuário, com demanda de antibioticoterapia – presente/ ausente.
- Quimioterapia - tratamento sistêmico por drogas antiblásticas ingeridas ou injetadas, após a cirurgia: sim / não.
- Antecedentes músculo-esqueléticos – relato de alterações ósseas, articulares, tendinosas ou musculares na articulação do ombro anterior à cirurgia, categorizada em: fraturas, luxações, bursite, tendinite, dor articular sem causa conhecida e ausente.

**5.5. Técnicas, testes e/ou exames**

**5.5.1. Técnicas**

**5.5.1.1. Cinesioterapia**

A técnica de fisioterapia a ser utilizada é a cinesioterapia através de 19 exercícios combinando movimentos de flexão, extensão, abdução, adução, rotação interna e rotação externa dos membros superiores, isolados ou combinados, e relaxamento de acordo com o protocolo assistencial do serviço (Anexo 1) ^23-25^.

Os exercícios serão ministrados pela equipe de especializandas do serviço de fisioterapia do CAISM/UNICAMP, de acordo com a rotina do serviço, sob supervisão das fisioterapeutas responsáveis pelo serviço.

O grupo de cinesioterapia realizará os exercícios a partir do 1º dia de pós-operatório e as pacientes receberão o material informativo, contendo orientações de cuidados com membro e de exercícios para serem realizados em domicílio e serão orientadas a manter atividade livre com o membro acometido em suas atividades diárias. Após 48 horas da cirurgia, os exercícios passarão a ser realizados no Ambulatório de Fisioterapia do CAISM-UNICAMP, com sessões em grupo (5 a 20 mulheres) de 40 minutos de duração, 2 vezes por semana, por um período de 30 dias.

**5.5.1.2. Drenagem Linfática Manual**

A DLM é uma técnica complexa de massagem, com manobras específicas e suaves, que atuam sobre o sistema linfático superficial, visando drenar o excesso de líquido que se acumula no interstício, nos tecidos e dentro dos vasos. Além disso, a DLM estimula as anastomoses linfolinfáticas e direciona a linfa para o local desejado ^42^.

As manobras de DLM utilizadas serão:

- Ganglionar, realizada sobre cadeias ganglionares próximas à região operada: região axilar contralateral e região inguinal homolateral;
- Evacuação, realizada através de manobras suaves visam deslocar a linfa do braço edemaciado em direção às regiões previamente evacuadas pela manobra ganglionar.

No pós-operatório, enquanto estiverem internadas, as mulheres do grupo de DML realizarão exercícios para membros superirores a partir do 1º dia de pós-operatório e receberão o material informativo, contendo orientações de cuidados com membro e de exercícios para serem realizados em domicílio e serão orientadas a manter atividade livre com o membro acometido em suas atividades diárias.

Após 48 horas da cirurgia, iniciarão as sessões de DLM que serão realizadas pela pesquisadora, no Ambulatório de Fisioterapia do CAISM-UNICAMP, terão duração de 40 minutos, freqüência de 2 vezes por semana, por um período de 30 dias. A DLM será realizada em atendimento individual.

Ambos os grupos assistirão às palestras da equipe multidisciplinar (psicologia, enfermagem e assistente social), que compõe o Programa de Reabilitação para pacientes operadas por câncer de mama.

**5.5.2. Testes**

Os testes utilizados neste estudo serão a goniometria e a cirtometria realizados por uma fisioterapeuta previamente treinada, sem conhecimento do grupo ao qual a paciente foi alocada.

A goniometria será utilizada para medir a amplitude de movimento da articulação do ombro, em graus. O aparelho utilizado será um goniômetro (marca CARCI), que é um círculo completo (0 a 360°), que acompanha o arco de movimento. Para realizar a goniometria o sujeito permanecerá com a região a ser avaliada despida, com movimentação ativo-livre do ombro e com bom alinhamento postural.

Na flexão do ombro, o movimento será realizado levando o braço para frente, com a palma da mão voltada medialmente paralela ao plano sagital, em decúbito dorsal. Na abdução do ombro, o movimento será realizado levando o braço lateralmente ao corpo, com palma da mão voltada anteriormente paralela ao plano frontal, em decúbito lateral.

A cirtometria será realizada para medir a circunferência do membro superior, em centímetros. O instrumento utilizado será uma fita métrica flexível (marca Hoachstmass), realizada a 7,5 centimetros acima e abaixo da prega do cotovelo, na articulação metacarpofalangiana e no processo estilóide da ulna.

**5.5.3. Exames**

As compensações do sistema linfático serão avaliadas através da linfocintilografia. As mulheres serão selecionadas e randomizadas no ambulatório de oncologia mamária do CAISM/UNICAMP com até um mês de antecedência da cirurgia, pela pesquisadora responsável e aplicado o Termo de Consentimento Livre e Esclarecido (ANEXO 2). Após concordar em participar do estudo, será feita a aleatorização e realizada a avaliação pré-operatória. Em seguida será agendado o exame de linfocintilografia que deverá ser realizado num período máximo de 30 dias antes da cirurgia.

A linfocintilografia será realizada no Serviço de Medicina Nuclear do Hospital das Clínicas da UNICAMP em cada avaliação – pré-operatório e 60 dias após a cirurgia. Será feita uma injeção intradérmica de fitato-99mTc no dorso de mão. As imagens serão obtidas após 10 minutos, 1 e 2 horas por uma câmara de cintilação APEX SP6 da Elscint (HIFA) equipada com colimador de baixa e média resolução. A dose utilizada do radiofármaco é de 1 a 2 mCi (37MBq) no membro ipsilateral e contra-lateral a cirurgia, os quais estarão com restrição de movimentos durante a realização do exame.

Os exames serão realizados por um técnico especializado e os laudos serão emitidos por dois avaliadores independentes, com experiência comprovada, e em caso de dúvidas ou discrepâncias um terceiro avaliador será requisitado. Constará da descrição estática e dinâmica dos resultados obtidos, sendo classificados segundo tabela abaixo (Rezende, 2008):

| **Velocidade** |  | **Grau de captção** |  |
| --- | --- | --- | --- |
| I | Linfonodos visíveis aos 10 minutos | A | Acentuado |
| II | Linfonodos visíveis após uma hora | B | Moderado |
| III | Linfonodos visíveis após duas horas | C | Discreto |
| IV | Linfnodos não visíveis | D | Ausente |

E então a função linfática será categorizada em:

- Normal: Ia – II a
- Alterada: II b – III c
- Ausente: IV d

A linfocintiligrafia será avaliada mediante as imagens obtidas nos 10 minutos, 1 e 2 horas após a injeção do radiofármaco. Os laudos serão descritos respeitando os objetivos específicos deste estudo.

**5.6. Instrumentos para coleta de dados**

O instrumento utilizado para coleta de dados será uma ficha de avaliação fisioterápica, preparada pela investigadora. A parte correspondente a identificação da paciente será destacada a fim de preservação do sigilo (ANEXO 3).

**5.7. Coleta de dados**

Os dados pré-operatórios foram coletados no Ambulatório de Fisioterapia em média XX dias antes da cirurgia. O exame de linfocintilografia foi realizado no pré-operatório XX dias antes da cirurgia e após XX dias da cirurgia.

**7. Controle de qualidade**

Fisioterapeutas especializandas do serviço de Fisioterapia do CAISM serão treinadas para aplicação do protocolo de exercícios nas pacientes pela pesquisadora responsável. A realização de exercícios mediante um protocolo já é rotina no serviço de Fisioterapia do CAISM – UNICAMP.

A DLM será realizada pela pesquisadora, duas vezes por semana no Ambulatório de Fisioterapia do CAISM/UNICAMP.

Uma fisioterapia do serviço de Fisioterapia do CAISM/UNICAMP será responsável pela coleta dos dados e para preenchimento da ficha de avaliação, para manutenção da imparcialidade dos dados obtidos.

A linfocintilografia será realizada no serviço de Medicina Nuclear do Hospital das Clínicas – UNICAMP, e terá um docente responsável pela emissão dos laudos e será realizada sempre no mesmo equipamento.

**8. Processamento e análise dos dados**

**8.1. Processamento de dados**

As fichas de avaliação serão submetidas a revisão com relação à qualidade e legitimidade das informações. Os dados serão codificados e duplamente digitados, utilizando-se o programa Excel, fazendo-se, então, limpeza dos dados, e obtendo-se o arquivo final a ser utilizado para análise, armazenados com cópia em local seguro.

**8.2. Análise dos dados**

Os dados serão avaliados descritivamente através do cálculo de freqüências absolutas (n) e relativas (%) para variáveis categóricas e através de média, mediana e desvio-padrão para as variáveis contínuas.

A homogeneidade dos grupos será analisada através do teste de Qui-quadrado ou Exato de Fisher, para variávies nominais e ordinais e do T-Student ou Mann-Whitney para as variáveis discretas. Para a comparação da evolução das medidas de ângulos e demais variáveis contínuas nos grupos será utilizado a MANOVA para medidas repetidas.

    O nível de significância assumido no trabalho é de 5%. O software utilizado para análise será o SAS, versão 9.1.3 (SAS Institute Inc., Cary, NC, USA, 2002-2003).

**9. Apresentação dos dados**

Tabela 1 – Características sócio-demográficas da amostra nos grupos de exercício (G1) e drenagem linfática manual (G2)

|  | G1  n=40 | Grupo 2  n=40 | P |
| --- | --- | --- | --- |
| Idade (anos) |  |  |  |
| IMC (kg/alt^2^) |  |  |  |
| Tipo de cirurgia  MRH  MRMP  MRMM |  |  |  |
| N^o^ de linfonodos comprometidos |  |  |  |
| N^o^ de linfonodos dissecados |  |  |  |
| Estadiamento cirúrgico  IIa  IIb  IIIa  IIIb  IIIc  IV |  |  |  |
| Infecção da ferida cirúrgica  Sim  Não |  |  |  |
| Quimioterapia  Sim  Não |  |  |  |

**Tabela 2** - Complicações pós-operatórias nos grupos de exercício (G1) e drenagem linfática manual (G2)

| **Variáveis** | **G1** | **G2** | **P** |
| --- | --- | --- | --- |
| Permanência do dreno (dias) |  |  |  |
| Seroma  Sim  Não |  |  |  |

**Tabela 3 -** Dados da função linfática pré-operatória, 30 e 60 dias pós-operatório segundo o grupo

|  | Pré-operatório | | 30 dias | | 60 dias | | valor p |
| --- | --- | --- | --- | --- | --- | --- | --- |
|  | G1 | G2 | G1 | G2 | G1 | G2 |  |
| Normal |  | |  | |  | |  |
| Alterado |  | |  | |  | |  |
| Ausente |  | |  | |  | |  |

**10. Considerações Éticas**

A realização deste estudo será baseada na Declaração de Helsinque (2001) e na Resolução 196/96 ^43^. O projeto será encaminhado para aprovação da Comissão de Pesquisa do Departamento de Tocoginecologia e pela CEP – Comissão de Ética em Pesquisa da FCM/ UNICAMP.

Será lido um termo de consentimento às mulheres, explicando sobre o caráter do estudo e esclarecendo sobre os riscos, os benefícios de sua participação e o sigilo em relação à fonte das informações, sendo garantido o direito à não participação sem qualquer tipo de prejuízo na sua assistência na instituição. Todos os informes constam no Termo de Consentimento Livre e Esclarecido (ANEXO 2) que será assinado pelas mulheres que aceitarem participar do estudo. Este consentimento será obtido pela pesquisadora, na primeira avaliação, realizada no Ambulatório de Oncologia do CAISM-UNICAMP, no momento da seleção dos sujeitos.

A literatura tem mostrado que a reabilitação física de mulheres submetidas à cirurgia de mama por neoplasia maligna é fator relevante para se evitar complicações imediatas ou tardias. A forma como a reabilitação foi preconizada neste protocolo, não oferece riscos ou prejuízos para a mastectomizada, pois em ambos os grupos as mulheres serão acompanhadas no pós-operatório e orientadas a realizar exercícios domiciliares.

**10. Referências Bibliográficas**

1. Brasil. Ministério da saúde.[INCA] Instituto Nacional de Câncer [on line]. 2008. Disponível em <http://www.inca.gov.br [11 Mar 2009]

2.Holmes CE, Muss HB. Diagnosis and Treatment of Breast Cancer in the Eldery. CA Cancer J Clin 2003;53:227-44.

3. Warren AG, Brorson h, Borud LJ, Slavin SA. Lymphedema – A comprehensive review. Annals of Plastic Surgery 2007; 59(4): 464-472

4. Clark B, Sitzia J, Harlow W. Incidence and risk of arm oedema following treatment for breast cancer: a three-year follow-up study. QJM 2005;98(5):343-8.

5. Petreck JA, Heelean MC. Incidence of Breast Carcinoma-Related Lymphedema. Cancer Suppl 1998;83(12):2776-81.

6. [Edwards MJ](http://www.ncbi.nlm.nih.gov/sites/entrez?Db=pubmed&Cmd=Search&Term="Edwards MJ"%5BAuthor%5D&itool=EntrezSystem2.PEntrez.Pubmed.Pubmed_ResultsPanel.Pubmed_DiscoveryPanel.Pubmed_RVAbstractPlus), [Whitworth P](http://www.ncbi.nlm.nih.gov/sites/entrez?Db=pubmed&Cmd=Search&Term="Whitworth P"%5BAuthor%5D&itool=EntrezSystem2.PEntrez.Pubmed.Pubmed_ResultsPanel.Pubmed_DiscoveryPanel.Pubmed_RVAbstractPlus), [Tafra L](http://www.ncbi.nlm.nih.gov/sites/entrez?Db=pubmed&Cmd=Search&Term="Tafra L"%5BAuthor%5D&itool=EntrezSystem2.PEntrez.Pubmed.Pubmed_ResultsPanel.Pubmed_DiscoveryPanel.Pubmed_RVAbstractPlus), [McMasters KM](http://www.ncbi.nlm.nih.gov/sites/entrez?Db=pubmed&Cmd=Search&Term="McMasters KM"%5BAuthor%5D&itool=EntrezSystem2.PEntrez.Pubmed.Pubmed_ResultsPanel.Pubmed_DiscoveryPanel.Pubmed_RVAbstractPlus). The details of successful sentinel lymph node staging for breast cancer [Am J Surg.](javascript:AL_get(this, 'jour', 'Am J Surg.');) 2000 Oct;180(4):257-61

7. Freitas JR, Ribeiro LFR, Taia L, Kajita D, Fernandes MV, Queiroz GS. Linfedema em pacientes submetidas à mastectomia radical modificada. Rev Bra. Ginecol Obstet 2001; 23(4): 205-8.

- 8.Liljegren G, Holmberg L. Arm mobility after sector resection and axillary dissection with or without postoperative radiotherapy in breast cancer stage I. Results from a randomised trial. Uppsala-Orebro Breast Cancer Study Group. Eur J Cancer 1997;33:193-9.

9.Celebioglu F, Perbeck L, Frisell J, Grondal E, Svensson L, Danielsson R. Lymph Drainage Studied by Lymphoscintigraphy in the Arms after Sentinel Node Biopsy Compared with Axillary Lymph Node Dissection Following Conservative Breast Cancer Surgery. Acta Radiologica 2007;48(5):488- 495

10.Kiel KD, Rademacker AW. Early stage breast cancer: arm edema after wide excision and breast irradiation. Radiology 1996;198: 279-83.

11. Hayes SC, Janda M, Cornish B, Battistutta D,Newman B. Lymphedema after breast cancer: incidence, risk factors, and effect on upper body function. J Clin Oncol 2008; 26:3536-3542

12. Stanton AWB, Modi S, Mellor RH, Peters AM, Svensson WE, Levick JR, Mortimer PS. A quantitative lymphoscintigraphic evaluation of lymphatic function in the swollen hands of women with lymphoedema following breast cancer treatment. Clinical Science (2006) 110, 553–561

13. Kim C, Li B, Papaiconomou C, Zakharov A, Johnston M. Functional impacto of lymphangiogenesis on fluid transport after lymph node excision. Lymphology 2003;36:111-9.

14. Foldi E, Foldi M, Clodius L. The Lymphedema chaos: a lancet. Ann Plast Surg 1989;22(6):505-15.

15. [Schmitz KH](http://www.ncbi.nlm.nih.gov/sites/entrez?Db=pubmed&Cmd=Search&Term="Schmitz KH"%5BAuthor%5D&itool=EntrezSystem2.PEntrez.Pubmed.Pubmed_ResultsPanel.Pubmed_DiscoveryPanel.Pubmed_RVAbstractPlus), [Troxel AB](http://www.ncbi.nlm.nih.gov/sites/entrez?Db=pubmed&Cmd=Search&Term="Troxel AB"%5BAuthor%5D&itool=EntrezSystem2.PEntrez.Pubmed.Pubmed_ResultsPanel.Pubmed_DiscoveryPanel.Pubmed_RVAbstractPlus), [Cheville A](http://www.ncbi.nlm.nih.gov/sites/entrez?Db=pubmed&Cmd=Search&Term="Cheville A"%5BAuthor%5D&itool=EntrezSystem2.PEntrez.Pubmed.Pubmed_ResultsPanel.Pubmed_DiscoveryPanel.Pubmed_RVAbstractPlus), [Grant LL](http://www.ncbi.nlm.nih.gov/sites/entrez?Db=pubmed&Cmd=Search&Term="Grant LL"%5BAuthor%5D&itool=EntrezSystem2.PEntrez.Pubmed.Pubmed_ResultsPanel.Pubmed_DiscoveryPanel.Pubmed_RVAbstractPlus), [Bryan CJ](http://www.ncbi.nlm.nih.gov/sites/entrez?Db=pubmed&Cmd=Search&Term="Bryan CJ"%5BAuthor%5D&itool=EntrezSystem2.PEntrez.Pubmed.Pubmed_ResultsPanel.Pubmed_DiscoveryPanel.Pubmed_RVAbstractPlus), [Gross CR](http://www.ncbi.nlm.nih.gov/sites/entrez?Db=pubmed&Cmd=Search&Term="Gross CR"%5BAuthor%5D&itool=EntrezSystem2.PEntrez.Pubmed.Pubmed_ResultsPanel.Pubmed_DiscoveryPanel.Pubmed_RVAbstractPlus), [Lytle LA](http://www.ncbi.nlm.nih.gov/sites/entrez?Db=pubmed&Cmd=Search&Term="Lytle LA"%5BAuthor%5D&itool=EntrezSystem2.PEntrez.Pubmed.Pubmed_ResultsPanel.Pubmed_DiscoveryPanel.Pubmed_RVAbstractPlus), [Ahmed RL](http://www.ncbi.nlm.nih.gov/sites/entrez?Db=pubmed&Cmd=Search&Term="Ahmed RL"%5BAuthor%5D&itool=EntrezSystem2.PEntrez.Pubmed.Pubmed_ResultsPanel.Pubmed_DiscoveryPanel.Pubmed_RVAbstractPlus). Physical activity and lymphedema (the PAL trial): Assessing the safety of progressive strength training in breast cancer survivors. [Contemp Clin Trials.](javascript:AL_get(this, 'jour', 'Contemp Clin Trials.');) 2009;30(3):233-45.

16.Van Der Horst ChMAM, Kenter JAL, De Jong MT, Keeman JN. Shoulder function following early mobilization of the shoulder after mastectomy and axillary dissection. Neth J Surg 1985;37(4):105-8.

17. Guyton AC. Tratado de Fisiologia Médica. Rio de Janeiro: Guanabara Koogan, 1992.

18. Gashev AA, Zawieja DC. Physiology of human lymphatic contractility: a historical perspective. Lymphology 2001;34:124-34.

19. Lane K, Worsley D, Mckenzie D. Exercice and the Lymphatic System - Implications for breast-cancer survivors. Sport Med, 2005; 35(6):461-471

20.Clodius L. Minimizing secondary lymphedema from axillary dissection. Lymphology 2001;34:106-10.

21. [Wingate L](http://www.ncbi.nlm.nih.gov/sites/entrez?Db=pubmed&Cmd=Search&Term="Wingate L"%5BAuthor%5D&itool=EntrezSystem2.PEntrez.Pubmed.Pubmed_ResultsPanel.Pubmed_DiscoveryPanel.Pubmed_RVAbstractPlus). Efficacy of physical therapy for patients who have undergone mastectomies. A prospective study. [Phys Ther.](javascript:AL_get(this, 'jour', 'Phys Ther.');) 1985 Jun;65(6):896-900

22. Wingate L, Croghan I, Natarajan N, Michalek AM, Jordan C. Rehabilitation of the mastectomy patient: a randomized, blind, prospective study. Arch Phys Med Rehabil 1989; 70:21-4.

- 23. [de Rezende LF](http://www.ncbi.nlm.nih.gov/sites/entrez?Db=pubmed&Cmd=Search&Term="de Rezende LF"%5BAuthor%5D&itool=EntrezSystem2.PEntrez.Pubmed.Pubmed_ResultsPanel.Pubmed_DiscoveryPanel.Pubmed_RVAbstractPlus), [Franco RL](http://www.ncbi.nlm.nih.gov/sites/entrez?Db=pubmed&Cmd=Search&Term="Franco RL"%5BAuthor%5D&itool=EntrezSystem2.PEntrez.Pubmed.Pubmed_ResultsPanel.Pubmed_DiscoveryPanel.Pubmed_RVAbstractPlus), [de Rezende MF](http://www.ncbi.nlm.nih.gov/sites/entrez?Db=pubmed&Cmd=Search&Term="de Rezende MF"%5BAuthor%5D&itool=EntrezSystem2.PEntrez.Pubmed.Pubmed_ResultsPanel.Pubmed_DiscoveryPanel.Pubmed_RVAbstractPlus), [Beletti PO](http://www.ncbi.nlm.nih.gov/sites/entrez?Db=pubmed&Cmd=Search&Term="Beletti PO"%5BAuthor%5D&itool=EntrezSystem2.PEntrez.Pubmed.Pubmed_ResultsPanel.Pubmed_DiscoveryPanel.Pubmed_RVAbstractPlus), [Morais SS](http://www.ncbi.nlm.nih.gov/sites/entrez?Db=pubmed&Cmd=Search&Term="Morais SS"%5BAuthor%5D&itool=EntrezSystem2.PEntrez.Pubmed.Pubmed_ResultsPanel.Pubmed_DiscoveryPanel.Pubmed_RVAbstractPlus), [Gurgel MS](http://www.ncbi.nlm.nih.gov/sites/entrez?Db=pubmed&Cmd=Search&Term="Gurgel MS"%5BAuthor%5D&itool=EntrezSystem2.PEntrez.Pubmed.Pubmed_ResultsPanel.Pubmed_DiscoveryPanel.Pubmed_RVAbstractPlus). Two exercise schemes in postoperative breast cancer: comparison of effects on shoulder movement and lymphatic disturbance. [Tumori.](javascript:AL_get(this, 'jour', 'Tumori.');) 2006 Jan-Feb;92(1):55-61.

24. Amaral MTP, Teixeira LC, Derchain SFM, Nogueira MD, Pinto e Silva MP, Gonçalves AV. Orientação domiciliar: proposta de reabilitação física para mulheres submetidas à cirurgia por câncer de mama. Rev Cien Med 2005, 14(5): 405-13.

25. Silva, MPPinto e et al. Movimento do ombro após cirurgia por carcinoma invasor da mama: estudo randomizado prospectivo controlado de exercícios livres versus limitados a 90º no pós-operatório. *Rev. Bras. Ginecol. Obstet.* 2004; 26(2):125-130.

26. Bergmann A, Mattos IE, Koifman RJ, Koifman S. Morbidade após o tratamento para câncer de mama. 2000 Fisioterapia Brasil; 1: 101-9.

27. Wittlinger H. & Wittlinger G. (1992) Introduction to Dr Vodder’s Manual Lymph Drainage, Vol. 1: Basic Course, 4th ed. Haug Publishers, Heidelberg.

28. Hutzschenreuter P., Brümmer H. & Ebberfeld K. (1989) Experimental and clinical studies of the mechanisms of effect of manual lymph drainage therapy. Journal of Lymphology 13,62–64.

29. Leduc O, Bourgeois A. Bandagens: scintigraphic demonstration of its efficacy on colloidal protein reabsorption during muscle activity. International Congress of Lymphology 1989: 421-3.

30. [Franzeck UK](http://www.ncbi.nlm.nih.gov/sites/entrez?Db=pubmed&Cmd=Search&Term="Franzeck UK"%5BAuthor%5D&itool=EntrezSystem2.PEntrez.Pubmed.Pubmed_ResultsPanel.Pubmed_DiscoveryPanel.Pubmed_RVAbstractPlus), [Spiegel I](http://www.ncbi.nlm.nih.gov/sites/entrez?Db=pubmed&Cmd=Search&Term="Spiegel I"%5BAuthor%5D&itool=EntrezSystem2.PEntrez.Pubmed.Pubmed_ResultsPanel.Pubmed_DiscoveryPanel.Pubmed_RVAbstractPlus), [Fischer M](http://www.ncbi.nlm.nih.gov/sites/entrez?Db=pubmed&Cmd=Search&Term="Fischer M"%5BAuthor%5D&itool=EntrezSystem2.PEntrez.Pubmed.Pubmed_ResultsPanel.Pubmed_DiscoveryPanel.Pubmed_RVAbstractPlus), [Börtzler C](http://www.ncbi.nlm.nih.gov/sites/entrez?Db=pubmed&Cmd=Search&Term="Börtzler C"%5BAuthor%5D&itool=EntrezSystem2.PEntrez.Pubmed.Pubmed_ResultsPanel.Pubmed_DiscoveryPanel.Pubmed_RVAbstractPlus), [Stahel HU](http://www.ncbi.nlm.nih.gov/sites/entrez?Db=pubmed&Cmd=Search&Term="Stahel HU"%5BAuthor%5D&itool=EntrezSystem2.PEntrez.Pubmed.Pubmed_ResultsPanel.Pubmed_DiscoveryPanel.Pubmed_RVAbstractPlus), [Bollinger A](http://www.ncbi.nlm.nih.gov/sites/entrez?Db=pubmed&Cmd=Search&Term="Bollinger A"%5BAuthor%5D&itool=EntrezSystem2.PEntrez.Pubmed.Pubmed_ResultsPanel.Pubmed_DiscoveryPanel.Pubmed_RVAbstractPlus). Combined physical therapy for lymphedema evaluated by fluorescence microlymphography and lymph capillary pressure measurements. [J Vasc Res.](javascript:AL_get(this, 'jour', 'J Vasc Res.');) 1997;34(4):306-11.

31. Williams A.F., Vadgama A., Franks P.J. & Mortimer P.S. A randomized controlled crossover study of manual lymphatic drainage therapy in women with breast cancerrelated lymphoedema 2002; European Journal Of Cancer Care 11, 254–261

32. Ferrandez JC, Laroche JP, Serin D, Felix-Faure C, Vinot JM. Aspects lymphoscintigraphiques des effets du drainage lymphatique manuel. Journal des Maladies Vasculaires 1996, 21(5):283-9

33. Kafejian-Haddad AP, Perez JM, Castglioni ML, Miranda Júnior F, de Figueiredo LF. Lymphscintigraphic evaluation of manual lymphatic drainage for lower extremity lymphedema. Lymphology 2006, 39(1):41-8

34. Modi S, Stanton AWB, Mellor RH, Peters AM, Levick JR, Mortimer PS. Regional distribution of epifascial swelling and epifascial lymph drainage rate constants in breast cancer-related lymphedema. Lymphat Res Biol 2005,3:3-15

35. Szuba A, Shin WS, Strauss W, Rockson S. The third circulation: radionuclide lymphoscintigraphy in the evaluation of lymphedema. J Nucl Med 2003;44(1):43-57.

36. Bourgeois P, Leduc O, Leduc A. Imaging techniques in the management and prevention of posttherapeutic upper limb edemas. Cancer Suppl 1998;83(12):2805-13.

37. Svensson W, Glass DM, Bradley D, Peters AM. Meansurement of lymphatic function with technetium-99m-labelled polyclonal immunoglobulin. Eur J Nucl Med 1999; 26(5):504-10.

38. Hwang JH, Kwon JY, Lee KW et al. Changes in lymphatic function after complex physical therapy for lymphedema. Lymphology 1999;32:15-21.

39. O’Mahony S, Rose SL, Chilvers AJ, Ballinger JR, Solanki CK, Barber RW, Mortimer PS, Purushotham AD, Peters AM. Finding an optimal method for imaging lymphatic vessels of the upper limb. Eur J Nucl Med Mol Imaging 2004; 31:555–563

40. Campisi C, Boccardo F, Zilli A, Maccio A, Napoli F, FerreiraAzevedo W Jr, et al. Lymphedema secondary to breast cancer treatment: possibility of diagnostic

and therapeutic prevention. Ann Ital Chir 2002;73:493–8.

41. Rezende LF. Avaliação das compensações linfáticas no pós-operatório de mastectomia radical através da linfocintilografia. Campinas, SP , 2008.

Orientador : Maria Salete Costa Gurgel. Tese (Doutorado ) Universidade Estadual de Campinas. Faculdade de Ciências Médicas.

42. Camargo MC, Marx AG. Reablilitação física no câncer de mama, São Paulo: Editora Roca, 2000

43. Brasil. Ministério da saúde. Conselho Nacional da Saúde. – Resolução Nº196/96 sobre pesquisa envolvendoseres humanos. Inf. Epidem. SUS, v.2; 1996.

**11. Cronograma**

| Atividade | 1 | 2 | 3 | 4 | 5 | 6 | 7 | 8 | 9 | 10 | 11 | 12 | 13 | 14 | 15 | 16 | 17 | 18 | 19 | 20 |
| --- | --- | --- | --- | --- | --- | --- | --- | --- | --- | --- | --- | --- | --- | --- | --- | --- | --- | --- | --- | --- |
| Revisão bibliográfica contínua | X | X | X | X | X | X | X | X | X | X | X | X | X | X | X | X | X | X | X | X |
| Coleta de dados |  |  | X | X | X | X | X | X | X | X | X | X | X | X | X | X | X | X |  |  |
| Acompanhamento dos sujeitos |  |  | X | X | X | X | X | X | X | X | X | X | X | X | X | X | X | X | X |  |
| Coleta de dados – revisão de prontuários |  |  |  |  |  |  |  |  |  |  |  |  |  |  | X | X | X | X | X |  |
| Processamento e análise dos dados |  |  |  |  |  |  |  |  |  |  |  |  |  | X | X | X | X | X | X | X |
| Redação do trabalho |  |  |  |  |  |  |  |  |  |  |  |  | X | X | X | X | X | X | X | X |

**12. Orçamento**

**Material de consumo**

| **Recursos** | **Quantidade** | **Custo Unitário** | **Valor solicitado** |
| --- | --- | --- | --- |
| **Microcomputador** | **1** | **R$ 2.000,00** | **R$ 2.000,00** |
| **Impressora** | **1** | **R$ 1.000,00** | **R$ 1.000,00** |
| **Cartucho para impressão** | **2** | **R$ 100,00** | **R$ 200,00** |
| **Papel Sulfite** | **1000** | **R$ 0,01** | **R$ 20,00** |
| **Exame de linfocintilografia** | **420** | **R$ 137,70** | **R$ 57.834,00** |

**Serviço de terceiros**

| **Recursos** | **Quantidade** | **Custo Unitário** | **Valor solicitado** |
| --- | --- | --- | --- |
| **Auxiliar para coleta de dados** | **1** | **R$ 4.500,00 (R$ 300,00 mensais)** | **R$ 4.500,00** |
| **Tradutora para os artigos** | **1** | **R$ 1.500,00 (R$ 750,00 por artigo)** | **R$ 1.500,00** |

**Valor total: R$ 67.054,00 (**A ser solicitado à FAPESP)

**ANEXO 1**

**Protocolo de Exercícios**

***1º Exercício***

**A.** Em posição neutra dos ombros, dobrar e estender os cotovelos por 10 vezes.

**B.** Com os braços erguidos, dobrar e esticar os cotovelos por 10 vezes.

**C.** Com os braços erguidos na altura das orelhas, dobrar e esticar os cotovelos, por 10 vezes.


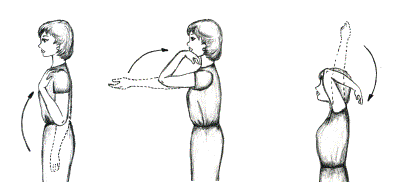


**FIGURA 1 – DESENHO REPRESENTATIVO DO 1º EXERCÍCIO**

***2º Exercício***

**A.** Esticar os braços ao lado do corpo, abrir e fechar as mãos e ao mesmo tempo, erguer os braços pela frente, até a altura das orelhas. Fazer 10 vezes.

**B.** Com a mesma posição anterior, abrir e fechar as mãos abrindo os braços lateralmente ao seu corpo. Faça 10 vezes.


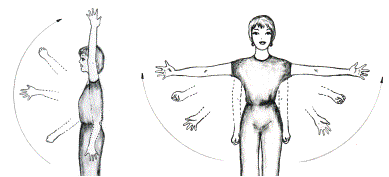


**FIGURA 2– DESENHO REPRESENTATIVO DO 2º EXERCÍCIO**

***3º Exercício***

Entrelaçar as mãos em frente à barriga e erguer os braços até as costas das mãos encostarem na testa. Faça 10 vezes.


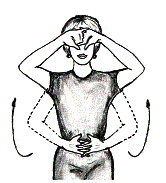


**FIGURA 3 – DESENHO REPRESENTATIVO DO 3º EXERCÍCIO**

***4º Exercício***

Com os braços abertos lateralmente ao corpo, dobrar e esticar os cotovelos. Faça 10 vezes.

Leve o cotovelo dobrado para trás e para frente, até que eles se encontrem anteriormente. Faça 10 vezes.


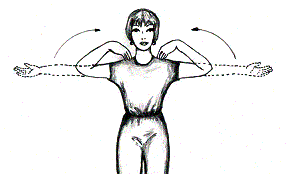


**FIGURA 4 – DESENHO REPRESENTATIVO DO 4º EXERCÍCIO**

***5º Exercício***

Cruzar as mãos atrás da nuca, abrir e fechar os cotovelos por 10 vezes.

Ao leva-los para trás, mantenha 20 segundos no máximo que conseguir.


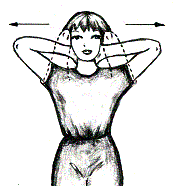


**FIGURA 5 – DESENHO REPRESENTATIVO DO 5º EXERCÍCIO**

***6º Exercício***

Colocar as mãos sobre os ombros, subir e abaixar os cotovelos, por 10 vezes.


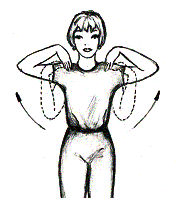


**FIGURA 6 – DESENHO REPRESENTATIVO DO 6º EXERCÍCIO**

***7º Exercício***

Com o uso de um bastão (cabo de vassoura), segure-o com os cotovelos estendidos, afastando-o e aproximando-o das nádegas. Faça 10 vezes.


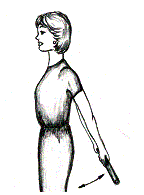


**FIGURA 7 – DESENHO REPRESENTATIVO DO 7º EXERCÍCIO**

***8º Exercício***

Na mesma posição anterior, subir o bastão até à cintura e descer novamente até às nádegas. Faça 10 vezes.

Rodar o corpo de um lado para o outro, mantendo 20 segundos em cada lado.


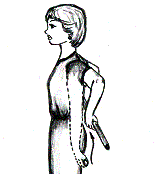


**FIGURA 8 – DESENHO REPRESENTATIVO DO 8º EXERCÍCIO**

***9º Exercício***

Segurar o bastão nas costas, com uma mão por cima do ombro e a outra por baixo, fazendo um movimento de vai e vem. Faça 10 vezes de cada lado.


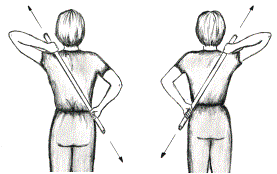


**FIGURA 9 – DESENHO REPRESENTATIVO DO 9º EXERCÍCIO**

1. **POSIÇÃO DEITADA DE COSTAS** – (COM AS PERNAS DOBRADAS)

***10º Exercício***

**A.** Abrir os braços ao lado do corpo e dobrar os cotovelos.

**B.** Levar as mãos em direção ao chão, e voltar apontando-as para cima. Faça 10 vezes, mantendo por 20 segundos cada vez.


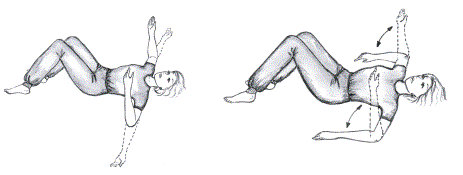


**FIGURA 10 – DESENHO REPRESENTATIVO DO 10º EXERCÍCIO**

***11º Exercício***

Arrastar os braços rente ao chão, em direção às orelhas, com a palma das mãos para cima. Faça 10 vezes.


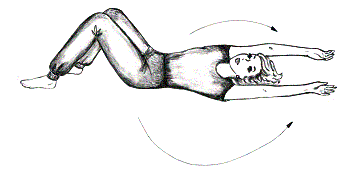


**FIGURA 11 – DESENHO REPRESENTATIVO DO 11º EXERCÍCIO**

***12º Exercício***

Entrelaçar as mãos em cima da barriga, esticar os braços até o seu limite. Faça 10 vezes, mantendo 20 segundos..


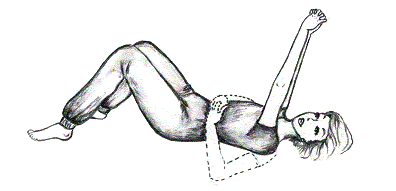


**FIGURA 12 – DESENHO REPRESENTATIVO DO 12º EXERCÍCIO**

***13º Exercício***

Abrir os braços, dobrar os cotovelos. Fazer o movimento de abrir e fechar os cotovelos, por 10 vezes (como se estivesse abrindo e fechando as asas). O cotovelo deverá se aproximar ao máximo do chão.


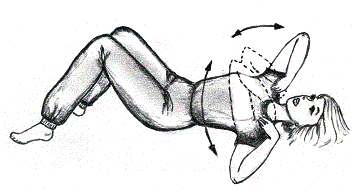


**FIGURA 13 – DESENHO REPRESENTATIVO DO 13º EXERCÍCIO**

1. **DEITADA DE LADO** - (NÃO DEITAR EM CIMA DO LADO OPERADO)

***14º Exercício***

Estender o braço ao longo do corpo (A), levá-lo em direção à orelha (B), permanecer durante 10 segundos nesta posição. Repetir o exercício 10 vezes.


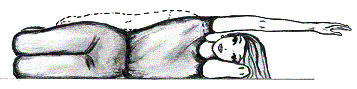


**FIGURA 14 – DESENHO REPRESENTATIVO DO 14º EXERCÍCIO**

***15º Exercício***

Estender o braço ao longo do corpo (A), levá-lo até à frente com o cotovelo esticado (B), abri-lo em direção às costas (C) e permanecer durante 20 segundos nesta posição. Repetir o exercício 10 vezes.


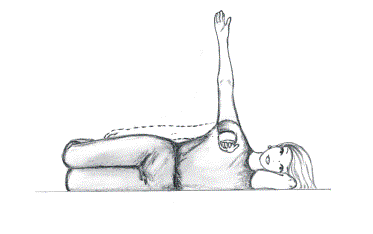


**FIGURA 15 – DESENHO REPRESENTATIVO DO 15º EXERCÍCIO**

***16º Exercício***

Estender o braço ao longo do corpo (A), levá-lo à frente com o cotovelo esticado (B), abri-lo diagonalmente ao corpo (C) e permanecer 20 segundos nesta posição. Repetir o exercício 10 vezes.


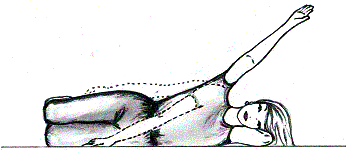


**FIGURA 16 – DESENHO REPRESENTATIVO DO 16º EXERCÍCIO**

**RELAXAMENTO**

Após a seqüência dos exercícios será feito um relaxamento com as mulheres deitadas, diminuindo as tensões física e mental, buscando promover sensação de repouso durante mais ou menos cinco minutos.


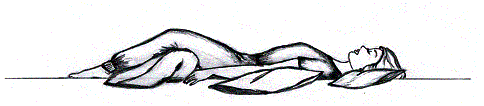


**FIGURA 17 – DESENHO REPRESENTATIVO DO RELAXAMENTO**

**ANEXO 2**

Comparação da cinesioterapia e da drenagem linfática manual nas complicações, compensações linfáticas e reabilitação do ombro no pós-operatório de câncer de mama

**TERMO DE CONSENTIMENTO LIVRE E ESCLARECIDO**

- **NOME:**
- **HC:**
- **Número de registro da pesquisa:**

**FUI INFORMADA QUE:**

A cirurgia realizada como forma de tratamento no câncer de mama pode trazer algumas complicações como a limitação do movimento do braço, acúmulo de líquido próximo a axila e inchaço do braço. Por este motivo, é necessário pesquisar soluções para prevenir ou ajudar nestes problemas.

Fazer exercício no pós-operatório dessa cirurgia é importante para que você não tenha problemas de movimento e inchaço do braço. Aqui no CAISM/ UNICAMP todas as pacientes já fazem esses exercícios há quase 20 anos. Porém, existe outra forma de fisioterapia chamada massagem de drenagem linfática manual que também pode contribuir para prevenção do inchaço do braço.

Esta pesquisa quer saber, qual a melhor abordagem da fisioterapia após a cirurgia de mama para que essas complicações sejam menores.

As participantes deste estudo serão divididas em dois grupos. Após a cirurgia, um grupo realizará exercícios, seguindo um protocolo e o outro grupo será submetido à drenagem lnfática do braço. Todas as participantes serão avaliadas antes da cirurgia e 60 dias depois, para análise da circulação do braço através de um exame chamado linfocintilografia que é um tipo de RX das veias do braço para tentar relacionar com o inchaço do braço. Para realização de exame é feita uma injeção no dorso de cada mão e então passara por avaliação do braço após 10 minutos, uma hora e duas horas. O exame não oferece riscos.

Qualquer um dos dois grupos terá o benefício de favorecer sua recuperação da mulher diminuindo possíveis problemas futuros e facilitar o retorno às atividades da vida diária.

Se eu quiser participar da pesquisa, me comprometo a comparecer 2 vezes por semana (às segundas e quartas -feiras) para realizar os exercícios ou as massagens e a realizar os exames necessários, mas a qualquer momento posso deixar de participar deste estudo sem que isso prejudique meu tratamento, realizando a fisioterapia que o serviço oferece normalmente com movimentação livre do braço da cirurgia no primeiro dia após a cirurgia e a não realização do exame. Sei também que serei sorteada e não poderei escolher qual dos grupos irei participar, caso eu aceite entrar no estudo.

Qualquer dúvida a respeito da pesquisa poderá ser esclarecida pelo pesquisador responsável. Podem ser pedidas informações junto ao Comitê de Ética de Pesquisa da UNICAMP.

Ninguém saberá do meu nome, mesmo que os resultados sejam publicados em revistas.

Ciente de tudo isto concordo em participar do estudo.

**DATA/____/____/____/**

**__________________________________**

**Mariana Maia Freire de Oliveira**

**Pesquisadora Responsável**

**🕾 Fisioterapia (19)35219428**

**__________________________________**

**Assinatura do paciente**

**Nome do paciente**

**COMITÊ DE ÉTICA DE PESQUISA DA UNICAMP**

**🕾(19)35218936**

**ANEXO 3**

# Ficha de avaliação

**1. IDENTIFICAÇÃO**

- Nome:

• Número de Registro da Pesquisa: /__/__/

- HC: *GRUPO DML* ڤ

*GRUPO CINESIOTERAPIA* ڤ

- Data da Avaliação:

**EXAME FÍSICO** Número de Registro da Pesquisa: /__/__/

*1. Goniometria do Membro Superior Ipsolateral à Cirurgia*

| **Goniometria** | **Pré** | **PO 30** | **PO 60** |
| --- | --- | --- | --- |
| **Flexão** |  |  |  |
| **Abdução** |  |  |  |

*2. Cirtometria do Membro Superior Ipsolateral à Cirurgia*

| **Cirtometria** | **Pré** | **PO 60** |
| --- | --- | --- |
| **Mão** |  |  |
| **Punho** |  |  |
| **Braço** |  |  |
| **Antebraço** |  |  |

- Dia da retirada do dreno: /___/___/___/
- Número de dias como dreno: /___/

*4. Seroma 1. Sim /__/ 2. Não /__/*

*5. Infecção 1. Sim /__/ 2. Não /__/*

6. Número de sessões realizadas: /___/

Número de faltas: /___/

*7. Linfocintilografia*

|  |  | **Pré** | **PO 60** |
| --- | --- | --- | --- |
| **Tempo de aparecimento dos vasos linfáticos** | **10 minutos** |  |  |
|  | **1 hora** |  |  |
|  | **2 horas** |  |  |
|  |  |  |  |
| **Tempo de aparecimento dos linfonodos regionais** | **10 minutos** |  |  |
|  | **1 hora** |  |  |
|  | **2 horas** |  |  |
|  |  |  |  |
| **Tempo de aparecimento no fígado** | **10 minutos** |  |  |
|  | **1 hora** |  |  |
|  | **2 horas** |  |  |
|  |  |  |  |

|  |  | **Pré** | **PO 60** |
| --- | --- | --- | --- |
| **Circulação colateral** | **Presente** |  |  |
|  | **Ausente** |  |  |
| **Refluxo para a derme** | **Presente** |  |  |
|  | **Ausente** |  |  |

**DADOS CIRÚRGICOS**

- **Data da Cirurgia:** /____/____/____/
- **Tipo de Cirurgia:** /____/

1-Mastectomia Radical Halsted

2-Mastecomia Radical Modificada Patey

3-Mastectomia Radical Modificada Madden

- **Mama Operada:** /____/

1-Direita

2-Esquerda

- **Estadiamento Clínico:** T /____/ N /____/ M /____ /
- **Estadiamento Cirúrgico:** T /____/ N /____/ M /____ /
- **Nível de retirada de linfonodos**: /____/
- **Número de linfonodos ressecados**: /____/
- **Nível de linfonodos positivos**: /____/
- **Peso:** /____/
- **Altura:** /____/
- **IMC: /___/**
- **Quimioterapia:** /____/ ciclos
- **Antecedentes músculo-esqueléticos**

Fratura /___/ luxação /____/ bursite /____/ tendinite /____/

dor sem causa conhecida /____/ ausente /____/
