# Supplementary material for: Long term effects of manual lymphatic drainage and active exercises on physical morbidities, lymphoscintigraphy parameters and lymphedema formation in patients operated due to breast cancer: A clinical trial
Source: PLoS One. 2018 Jan 5;13(1):e0189176. doi: 10.1371/journal.pone.0189176 (PMC5755747; doi:10.1371/journal.pone.0189176)
Supplement: S2 Text — (DOC) [file pone.0189176.s002.doc]

**Comparison of Kinesiotherapy and manual lymphatic drainage in the lymphatic compensation postoperative breast câncer**

1. **Participants selection**

This study will be dedicated to proceed a clinical trial involving women who will undergo radical mastectomy due to invasive breast carcinoma at the Prof. Dr. Jose´ Aristodemo Pinotti’s Women’s Hospital - Women’s Integrated Healthcare Center (CAISM/UNICAMP), Brazil.

All women meeting the inclusion criteria will be invited to participate and those who agree will assign to either the Manual Lymphatic Drainage (MLD) or the active exercise group. Matching criteria include clinical staging, age (10y), and body mass index (low body weight (<18.5Kg/m²); normal body weight (≥18.5 and ≤24.9Kg/m²); overweight (≥25 and ≤ 29.9Kg/m²) and obesity (≥ 30Kg/m²)). One week before the surgery, patients will be evaluated at the physical therapy outpatient clinic, during which personal and clinical data will be collected, UL circumference measurements, goniometry and lymphoscintigraphy will be performed. Sixty days after surgery, the women will be reevaluated.

**1.1. Inclusion criteria**

- First surgery for invasive breast carcinoma;
- Women who would perform unilateral mastectomy with lymph node dissection.
  1. **Exclusion criteria**
- Women who would perform immediate breast reconstruction;
- Women who before surgery had a difference in upper limbs circumference greater than two centimeters;
- women who had motor deficit or infection in the upper limb;
- Women who would perform sentinel lymph node biopsy;
- Women who underwent radiation therapy in ipsilateral to surgery in the preoperative;
- Women unable to comprehend the exercises proposed.

1. **Techniques, tests and / or exams**

**2.1. Techniques**

**2.1.1. Kinesiotherapy**

On the first postoperative day, all participants will receive informative leaflets about proper care for the operated limb in order to prevent trauma, infection, and to avoid activities that involve to much load and/or repetition; as well as daily active exercises to do at home. Furthermore, during the first month after surgery, patients will attend lectures delivered by the multidisciplinary team of psychologists, nurses, social service workers and dietitians, which compose the Rehabilitation Program for women undergoing breast cancer surgery.

After 48 hours of surgery, the exercises will be performed at the Physiotherapy Clinic of CAISM-UNICAMP, with group sessions (5 to 20 women) of 40 minutes duration, twice a week, for a period of 30 days.

The technique applied is the active exercise. The session will be divided into: 30% of the time – stretching of the scalene muscles, trapezius muscles, levator scapulae muscles, pectoralis major and minor muscles, rotator cuff muscles of the shoulder; 60% of the time – active assisted exercise and free active exercise for shoulder flexion, abduction, adduction, internal and external rotation of the UL alone or combined, followed by stretching of the deltoid, latissimus dorsi, rhomboids and pectoralis muscles; and 10% of the time-relaxation, according to treatment protocol of the service. Exercises will be done under supervision of the physical therapy team at CAISM/UNICAMP.

**2.1.2. Manual Lymphatic Drainage (MLD)**

Forty-eight hours after surgery, the women integrating this group will begin individual 40-minute MLD sessions, twice a week, for 30 days. Sessions will be conducted by three experienced physical therapists. MLD technique involved: (a) evacuation of lymph nodes in the contralateral axillary region and ipsilateral inguinal region, and (b) reabsorption performed by gentle massage strokes, firstly toward the axillo-axillary and axillo-inguinal anastomoses and, subsequently, on the limb ipsilateral to surgery. These manoeuvres aim to dislocate lymph from the UL toward the regions previously evacuated through lymph node massage.

**2.2. Tests**

- - 1. The circumference of the upper limb measure will be assessed in centimeters (cm) with a flexible measuring tape (trade name: Hoachstmass) at four predetermined points: (1) 7.5 cm above the elbow crease; (2) 7.5 cm below the elbow crease; (3) at the metacarpo-phalangeal joint; and (4) at the ulnar styloid process. To record the natural difference between limbs, the UL circumference will be assessed bilaterally before surgery.

For diagnosis of lymphedema, enlargement (difference) of 2 cm or more in arm circumference at any point compared with the contralateral limb will be considered of clinical significancy.

- - 1. Shoulder range of motion (ROM) will be evaluated by shoulder goniometry, using a universal goniometer graded in degrees. Maximum ROM will be considered 180o for movements of flexion and abduction (Marques, 1997). Shoulder flexion will be assessed in the supine position. The stationary arm of the goniometer will be centered along the midaxillary line of the trunk, pointing towards the femoral greater trochanter. The moving arm of the goniometer will be parallel to the longitudinal aspect of the humerus, pointing toward the lateral epicondyle. Goniometer axis will be centered close to the acromium process. The patient should perform an active movement, elevating the arm as far as possible, with the palm of the hand facing medially, parallel to the sagittal plane (Marques, 1997).

Shoulder abduction will be assessed in the side-lying position. The stationary arm of the goniometer will be centered over the axillary line posterior to the trunk. The moving arm of the goniometer will be centered over the posterior surface of the arm, facing the dorsal region of the hand. The woman should perform an active movement, elevating the arm laterally from the trunk as far as possible, with the palm of the hand facing medially.

- - 1. Inspection and palpation of surgical wound - Wound inspection will be performed in each session to identify signs of dehiscence or scarring due to infection. Wound dehiscence was defined as the separation of the surgical wound edges. Surgical site infection was defined as the presence of transudate, swelling, redness evaluated by inspection and hyperthermia assessed by palpation (Marques et al, 2011). In addition, the patient will be asked about the use of antibiotic therapy.

The presence of seroma will be evaluated by palpation and the patient will be asked about the outcome of needle aspiration after drain removal.

**2.3. Exam**

The lymphoscintigraphy will be performed immediately before surgery and 60 days after surgery at the Nuclear Medicine Service. One mCi (37 MBq) of 99mTc-dextran will be administered by intradermal injection in the dorsum of each hand. Imaging of the upper limbs (UL) in the anterior view, with the patient supine with elbows semi-flexed and supported hands on the trunk, will be performed after 10 minutes and after 1 and 2 hours with a scintillation camera (Elscint APEX SP6 and Millenium GE) equipped with a low-energy and high resolution collimator.

The images will be analyzed and two experienced nuclear physicians will issue the medical reports. Should there be any doubt or divergence, a third evaluator will be requested. Visual criteria analysis of lymphoscintigraphy findings of the UL was previously published by a study conducted at our service. This analysis included the velocity visualization of axillary lymph nodes (LN) and degree uptake in axillary LN, classified according to Table 1.

| Velocity visualization of axillary  lymph nodes | | Degree uptake in axillary lymph nodes | |
| --- | --- | --- | --- |
| I | At 10 minutes | A | Intense |
| II | After one hour | B | Moderate |
| III | After two hours | C | Mild |
| IV | Not visualized | D | Absent |

The presence or absence of radiopharmaceutical absorption by the liver, collateral circulation, and dermal backflow will also be analyzed. Liver absorption will be classified as present when radiopharmaceutical uptake by the liver was visualized in any image; collateral circulation will be classified as present when there was drainage of radioactive tracer by a lateral lymphatic vessel or uptake in an epitrochlear lymph node; and dermal backflow will be classified as present when there was dermal dispersion of the radiopharmaceutical.

**3.3. Data Collection**

Assessed for eligibility

Excluded (n = 20):

Preoperative evaluation

Exercise group

MLD group

Postoperative evaluation

Postoperative evaluation
